# A multifunctional composite hydrogel as an intrinsic and extrinsic coregulator for enhanced therapeutic efficacy for psoriasis

**DOI:** 10.1186/s12951-022-01368-y

**Published:** 2022-03-24

**Authors:** Jiangmei Xu, Hao Chen, Zhaoyou Chu, Zhu Li, Benjin Chen, Jianan Sun, Wei Lai, Yan Ma, Yulong He, Haisheng Qian, Fei Wang, Yunsheng Xu

**Affiliations:** 1 Department of Dermatovenerology, The Seventh Affiliated Hospital, Sun Yat-Sen University, Shenzhen, Guangdong People’s Republic of China; 2 Department of Dermatovenerology, The Third Affiliated Hospital, Sun Yat-Sen University, Guangzhou, Guangdong People’s Republic of China; 3 School of Basic Medical Sciences, School of Biomedical Engineering, Research and Engineering Center of Biomedical Materials, Anhui Provincial Institute of Translational Medicine, Hefei, Anhui People’s Republic of China; 4 Center for Digestive Disease, The Seventh Affiliated Hospital, Sun Yat-Sen University, Shenzhen, Guangdong People’s Republic of China

**Keywords:** Psoriasis, Immunoregulation, Hydrogel, Methotrexate, Transdermal delivery

## Abstract

**Background:**

Psoriasis is a chronic relapsing immunological skin disease characterized by multiple cross-talk inflammatory circuits which are relevantly associated with abnormal cross-reactivity between immune cells and keratinocytes (KCs). It may be inadequate to eradicate complicated pathogenesis only via single-mode therapy. To provide optimal combinatory therapeutics, a nanocomposite-based hydrogel was constructed by loading methotrexate (MTX) into ZnO/Ag to realize combined multiple target therapy of psoriasis.

**Results:**

In this composite hydrogel, ZnO hybrid mesoporous microspheres were utilized both as drug carriers and reactive oxygen species (ROS)-scavenging nanoparticles. A proper amount of Ag nanoparticle-anchored ZnO nanoparticles (ZnO/Ag) was functionalized with inherent immunoregulatory property. The experiments showed that ZnO/Ag nanoparticles could exhibit a self-therapeutic effect that was attributed to reducing innate cytokine profiles by inactivating p65 in proinflammatory macrophages and abrogating secretion of adaptive cytokines in KCs by downregulating ROS-mediated STAT3-cyclin D1 signaling. A preferable antipsoriatic efficacy was achieved via topical administration of this hydrogel on the imiquimod (IMQ)-induced psoriasis mice model, demonstrating the superior transdermal delivery and combined enhancement of therapeutic efficacy caused by intrinsic nanoparticles and extrinsic MTX.

**Conclusion:**

This composite hydrogel could serve as a multifunctional, nonirritating, noninvasive and effective transcutaneous nanoagent against psoriasis.

**Graphical Abstract:**

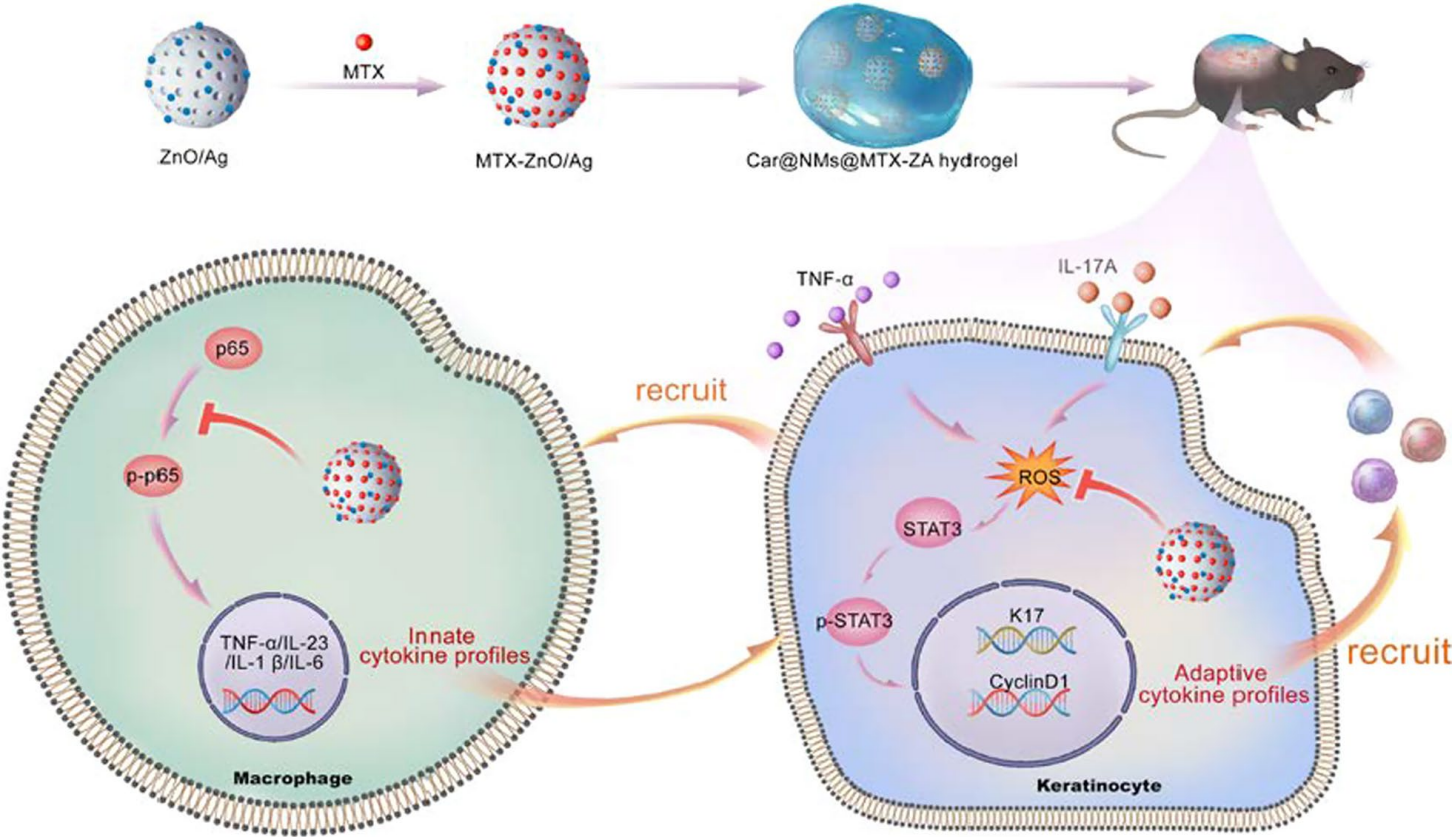

**Supplementary Information:**

The online version contains supplementary material available at 10.1186/s12951-022-01368-y.

## Background

Psoriasis is a chronic autoimmunological disorder that affects approximately 2% of the global population [1–3]. Mechanistically, intricate interactions between innate and adaptive immunities, with crucial roles for proinflammatory macrophages and "activated" keratinocytes (KCs), are the core of the pathogenesis of psoriasis [4–6]. Multiple inflammatory circuits mediated by these pathogenetic cells can disequilibrate the redox system of KCs, leading to inflammation and hyperproliferation of KCs [1, 7–9]. Therefore, targeting proinflammatory macrophages and "activated" KCs simultaneously could be emerged as an effective strategy for the treatment of psoriasis.

Methotrexate (MTX) is considered to be an immunosuppressive regulator involved in mediating the suppression of these pathogenetic cells. It has been systemically used in the treatment of moderate-to-severe psoriasis [10]. Currently, the introduction of hydrogel [11], liposome [12], polymers [13] and microneedles [14] nanocarriers, makes contributions to the transdermal delivery of MTX and other antipsoriatic drugs for the circumvention of overwhelming systemic adverse reactions in conventional therapy. However, desired therapeutic efficacy would be hindered by single-agent topical MTX therapy against multiple inflammatory loops in the pathogenesis of psoriasis. Thus, it could be further contributed to an increased risk of side effects and drug resistance caused by overused high-dose MTX. Recently, anti-inflammation of nanomaterials had been employed to inherent ability for the treatment of RA and psoriasis, such as cationic polymers [15], Au [16], FA-Ag [17] and manganese ferrite/ceria codecorated nanoparticles [18]. The hydrogel has been identified as the most competitive candidate for percutaneous treatment of skin diseases, which can be equipped with tunable functions via the incorporation of various nanoparticles due to its characteristics of good adhesiveness and skin retention [19–21]. Therefore, it’s an alternative strategy to fabricate a self-therapeutic multifunctional hydrogel nanocarrier and precisely controlled release of MTX in a specific manner for simultaneously inhibiting propagation of inflammatory circuits and enhancing low-dose MTX therapeutic effects to repress the aggravation of psoriasis.

**Scheme 1:**
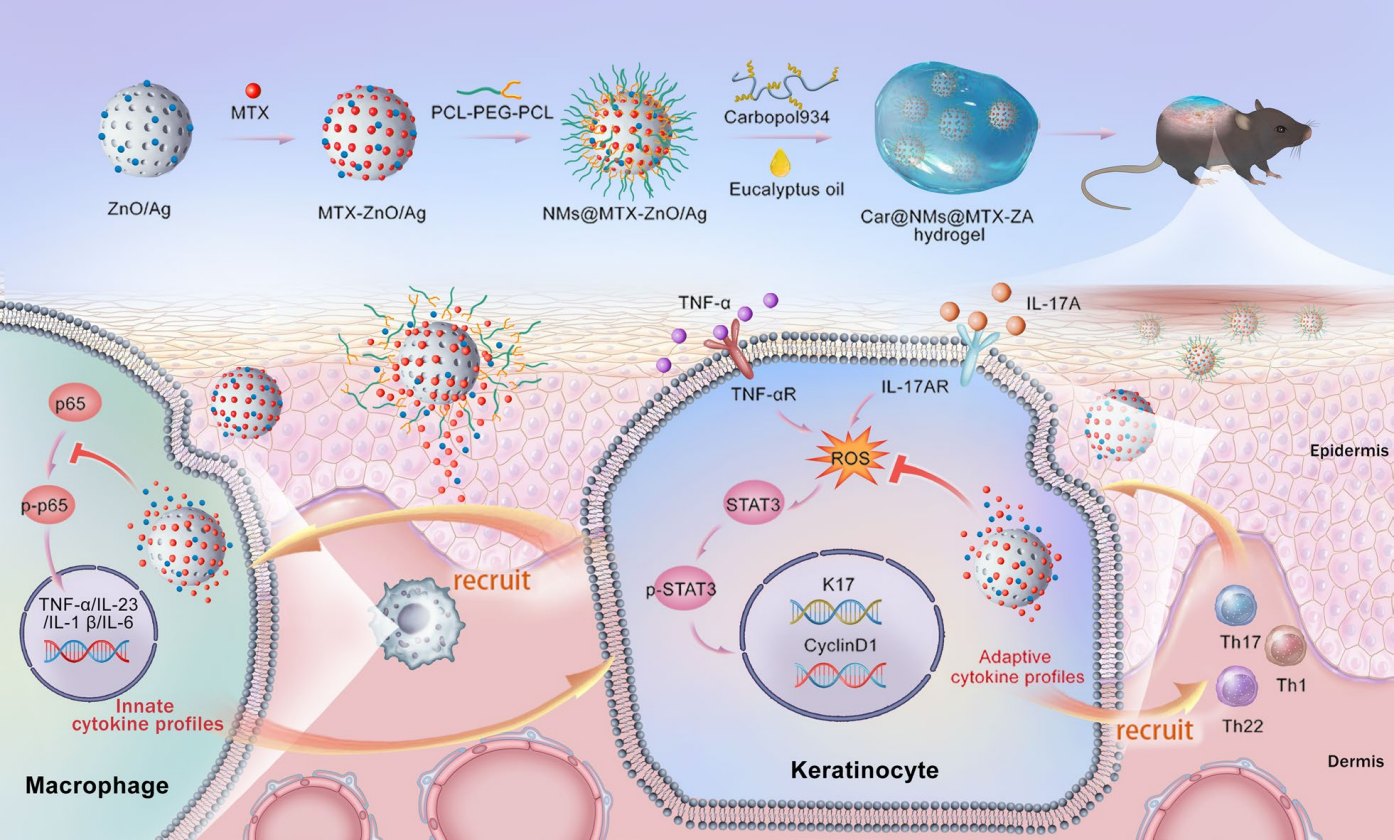
Schematic illustration of the Car@NMs@MTX-ZA hydrogel with intrinsic immunoregulation and ROS scavenging as well as extrinsic MTX for simultaneous anti-inflammatory and antiproliferative theranostics in psoriasis

Zinc oxide (ZnO) has been utilized in dermatological applications for many years due to its low toxicity and low cost [19]. In our previous work, we reported a good biocompatible AA-[Zn(OH)_4_]^2−^ (denoted as ZnO) hybrid mesoporous microspheres which can be served for drug delivery [20]. Herein, ZnO mesoporous microspheres were developed to incorporate MTX to achieve the sustainable release of MTX in the treatment of psoriasis. As illustrated in Scheme 1, ZnO/Ag (ZA) could qualify for self-therapeutic function. Ag nanoparticles were well dispersed in mesoporous microspheres, which endowed ZA with stable immunomodulatory activity to block the innate cytokine profiles by targeting macrophages. ZA could also regulate the STAT3-cyclin D1 signaling by eliminating reactive oxygen species (ROS), which restrained self-amplifying adaptive cytokine profiles. MTX-ZnO/Ag (MTX-ZA) nanoparticles were embellished with nanomicelles (NMs) and further embedded with Carbopol, denoted as Car@NMs@MTX-ZA hydrogel, which would show better therapeutic efficacy than MTX alone in imiquimod (IMQ)-induced psoriatic mice. In vivo and in vitro experiments, including qRT–PCR, ELISA and immunofluorescence analyses, were demonstrated. As expected, this composite hydrogel could optimize the therapeutic effects against psoriasis via synergistic multitherapy.

## Results and discussion

### Synthesis and charactrization of the multifunctional Car@NMs@MTX-ZA composite hydrogel

**Fig. 1:**
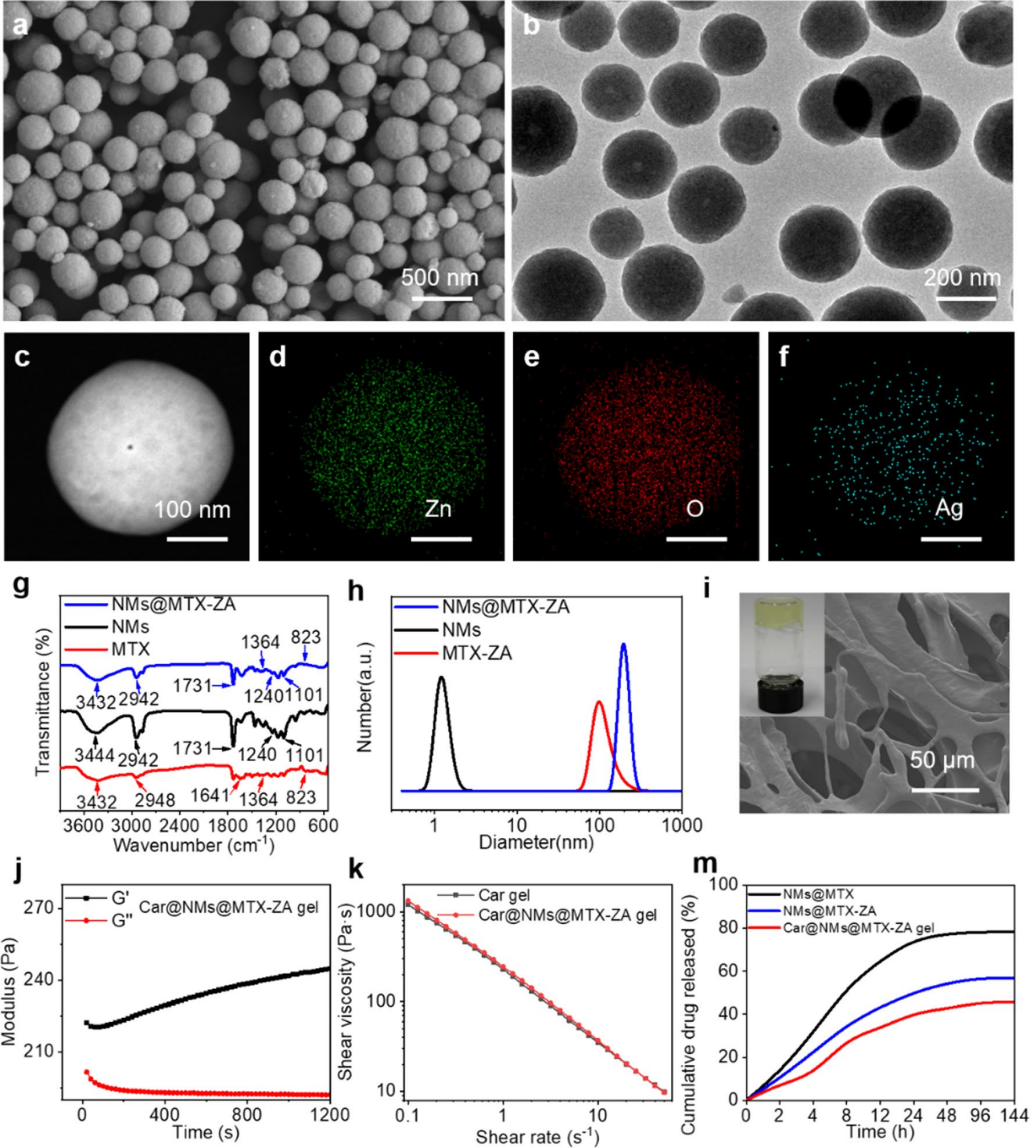
Characterization of the Car@NMs@MTX-ZA hydrogel. **a**, **b** SEM and TEM images of the ZnO/Ag hybrid microspheres. **c**–**f** STEM and elemental mapping images of the ZnO/Ag hybrid microspheres. **g** FT-IR spectra of NMs, MTX and NMs@MTX-ZA. **h** The size distributions of the NMs, MTX and NMs@MTX-ZA. **i** SEM image of porous structures and digital picture of the Car@NMs@MTX-ZA hydrogel. **j**–**k** Rheological properties of Car@NMs@MTX-ZA hydrogel. **m** In vitro release profiles of MTX from different formulations at 37 °C using phosphate buffered saline (pH = 7.4). NMs: PCL-PEG-PCL

As shown in Fig. 1a, b, the scanning electron microscope (SEM) and transmission electron microscopy (TEM) images showed that ZnO/Ag (ZA) hybrid microspheres with *ca.* 200 nm in diameter were prepared. Scanning transmission electron microscopy (STEM) and elemental mapping images indicated that the elements of Zn, Ag and O were homogeneously distributed in the overall microspheres (Fig. 1c–f). As previously described [13], the ^1^H-NMR spectra illuminated that the PCL-PEG-PCL-based nanomicelles (NMs) were synthesized (Additional file 1: Fig. S1a). FT-IR spectra (Fig. 1g) showed that the MTX was successfully introduced into the prepared nanomicelles. SEM image showed the spherical morphology of NMs@MTX-ZA (Additional file 1: Fig. S1b). The dynamic light scattering (DLS) and zeta potential of NMs@MTX-ZA exhibited an average size distribution of 202 nm and − 19.63 mV (Fig. 1h and Additional file 1: Fig. S2). After NMs@MTX-ZA was introduced into Carbopol, the obtained Car@NMs@MTX-ZA hydrogel showed a darker shade of yellow in appearance compared with the blank Carbopol hydrogel, and their typical pore structures were recorded by SEM (Fig. 1i and Additional file 1: Fig. S3). The curve of G′/G″ and viscosity measurements showed this hydrogel was successfully synthesized. Additionally, energy dispersive spectroscopy (EDS) and elemental mapping indicated that ZA was homogenously distributed in the as-prepared hydrogel (Fig. 1j–k, Additional file 1: Figs. S4 and S5).

In this hydrogel, the sustained release behavior of MTX in MTX-ZA was expected. The loading capacities of MTX in NMs@MTX-ZA and NMs@MTX were 22.95% and 16.86%, respectively. In vitro release studies were conducted to evaluate the release of MTX from various formulations in phosphate buffered saline at pH = 7.4. Approximately 74% free MTX in NMs@MTX was released within 24 h, nearly 50% was produced from NMs@MTX-ZA, and only 40.61% was released from the Car@NMs@MTX-ZA hydrogel (Fig. 1m and Additional file 1: Fig. S6a, b). Consistent with our anticipation, the Car@NMs@MTX-ZA hydrogel exhibited an optimal sustained release manner of MTX. This was primarily attributed to the controllable release performance of ZA [19].

### Self-therapeutic ZnO/Ag nanoparticles effectively dampened the innate cytokine profiles by restraining the phosphorylation of p65 in proinflammatory macrophages

Ag nanoparticles (Ag NPs) have immunomodulatory properties by elaborately controlling their content [17, 21]. In this work, AA-[Zn(OH)_4_]^2−^ (denoted as ZnO) hybrid mesoporous microspheres loaded with different amounts of Ag nanoparticles (0.01%, 0.1% and 1 wt%) were prepared according to the protocol reported by our group [22]. Their actual amount of Ag was determined by inductively coupled plasma optical emission spectroscopy (ICP-OES), as shown in Additional file 1: Table S1. The ICP-OES results of Ag^+^ ions released from them at 24 h and CCK-8 showed that the ZA with 0.1 wt% of Ag might have better anti-inflammatory activity with an ultralow concentration of Ag^+^ (approximately 0.20 ppb) and no cell cytotoxicity (Additional file 1: Figs. S7 and S8) [17, 23].

**Fig. 2:**
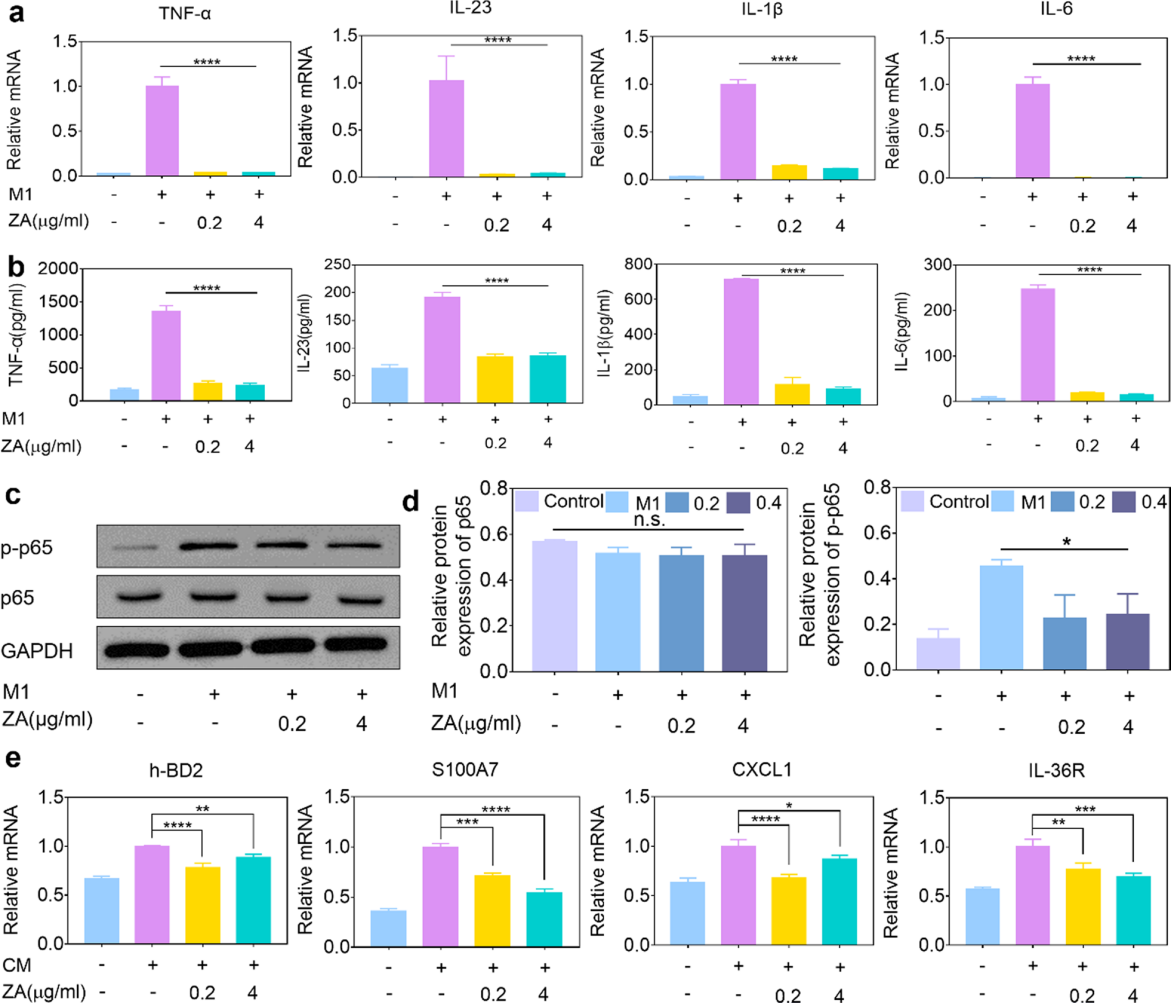
ZA attenuated innate cytokine profiles by reducing the phosphorylated form of p65. **a** mRNA and **b** protein levels of innate cytokine profiles from M1 cells measured by quantitative PCR and ELISA. **c**, **d** Quantitative analysis of the activation of phospho-p65 in M1 cells by western blotting. **e** Innate cytokine profiles expression in HaCaT cells with or without CM co-culture, analyzed by quantitative PCR. n = 3, mean ± SEM, n.s. (not significant), ^*^p < 0.05, ^**^p < 0.01, ^***^p < 0.001, ^****^p < 0.0001 vs. M1-treated group, M1: LPS and IFN-γ, ZA: 0.1 wt% ZnO/Ag, CM: conditional medium

According to the above data, we hypothesized that 0.1 wt% ZA could disrupt the induction of innate inflammatory cytokines, such as TNF-α, IL-23, IL-1β, and IL-6, in proinflammatory macrophages via limiting phosphorylated p65 (p-p65). To verify this hypothesis, cellular inflammatory models were employed in vitro. Nile red (NR) was used as a fluorescent substance to verify the endocytosis of THP-1 cells by confocal laser scanning microscopy (CLSM) (Additional file 1: Fig. S9). Then, we used LPS and IFN-γ to stimulate macrophages (denoted as M1 cells), and qRT–PCR analysis revealed that both 0.01 wt% ZA and 0.1 wt% ZA significantly suppressed the mRNA expression of psoriatic cytokines, including TNF-α, IL-23, IL-1β and IL-6 (Fig. 2a and Additional file 1: Fig. S10). Moreover, we further addressed whether ZA could prevent IL-23-driven macrophages from secreting the unique cytokines TNF-α and IL-17A [4]. Interestingly, IL-23 induced TNF-α mRNA expression in macrophages was downregulated by 0.01 wt% ZA and 0.1 wt% ZA, but IL-17A mRNA was only downregulated in the 0.1 wt% ZA-treated groups (Additional file 1: Fig. S11). Consistent with the data in Additional file 1: Fig. S7, 0.1 wt% ZA had a relatively better performance than 0.01 wt% ZA in suppressing the activation of proinflammatory macrophages, representing the optimal content of Ag. Therefore, 0.1 wt% ZA was chosen to perform all experiments. The protein expression of related innate cytokines from M1 cells was evaluated by ELISA analysis. As shown in Fig. 2b, ZA treatment groups markedly reduced the expression of the innate cytokines TNF-α, IL-23, IL-1β, and IL-6 in proinflammatory M1 cells. For LPS-induced RAW264.7 cells, 0.1 wt% ZA treatment exhibited a similar trend (Additional file 1: Fig. S12a, b). In addition, western blot analysis revealed that ZA could downregulate the phosphorylation of p65 in M1 cells (Fig. 2c, d), thus disrupting the induction of innate immunity. Moreover, innate cytokines generated from proinflammatory macrophages directly target effector KCs, leading to the production of inflammatory mediators, such as h-BD2, S100A7, CXCL1 and IL-36R [24]. These inotropic cytokines, in turn, recruit excessive macrophages infiltration to form an innate cytokine loop in psoriatic lesions [6, 25]. To further identify the comprehensive effects of ZA on the innate cytokine loop between macrophages and KCs, we respectively collected supernatants from M1 cells with and without ZA treatment as the conditional medium (CM), labeled as control, M1, M1 + ZA. HaCaT cells with CM co-culture were subjected to qRT–PCR analysis to monitor the inflammatory mediators released from HaCaT cells. Compared with the M1 condition co-culture group, the expression of inflammatory mediators, h-BD2, S100A7, CXCL1, and IL-36R, were inhibited in the M1 + ZA group, signifying the blockade of the autoimmune loop on KCs involved in the amplification of the innate immune response (Fig. 2e). These observations indicated that ZA, as an immunomodulator, could intrinsically block the operation of innate cytokine profiles crosstalk between macrophages and KCs.

### Self-therapeutic ZnO/Ag nanoparticles antagonized cyclin D1 expression and K17/cytokine autoimmune loop through eliminating ROS-induced STAT3 phosphorylation in psoriasiform keratinocytes

Parallelly, targeting "activated" KCs is another effective strategy against psoriasis. The cytokines TNF-α and IL-17A can stimulate hyperkeratinization and adaptive cytokines secretion in KCs by disturbing the oxidant-antioxidant system [26, 27]. Signal transducer and activator of transcription 3 (STAT3), as an essential transcription factor in KCs activated by detrimental ROS [26, 28, 29], can induce the expression of K17 and cyclin D1, which is responsible for the G1/S transition in the cell cycle, leading to the excessive proliferation of KCs [30, 31]. Meanwhile, K17 is recognized by autoreactive T cells, aggravating the K17/cytokine autoimmune loop in adaptive immunity of psoriasis [32]. Therefore, it is imperative to quench the production of ROS in pathogenetic KCs. As shown in Additional file 1: Fig. S13, the results of the ABTS test suggested that ZA and ZnO had comparable ROS scavenging abilities [33, 34]. Therefore, we speculated that ZA could inhibit cyclin D1 expression and block the self-amplifying K17/cytokine autoimmune loop on KCs by eliminating noxious ROS-induced STAT3 phosphorylation.

**Fig. 3:**
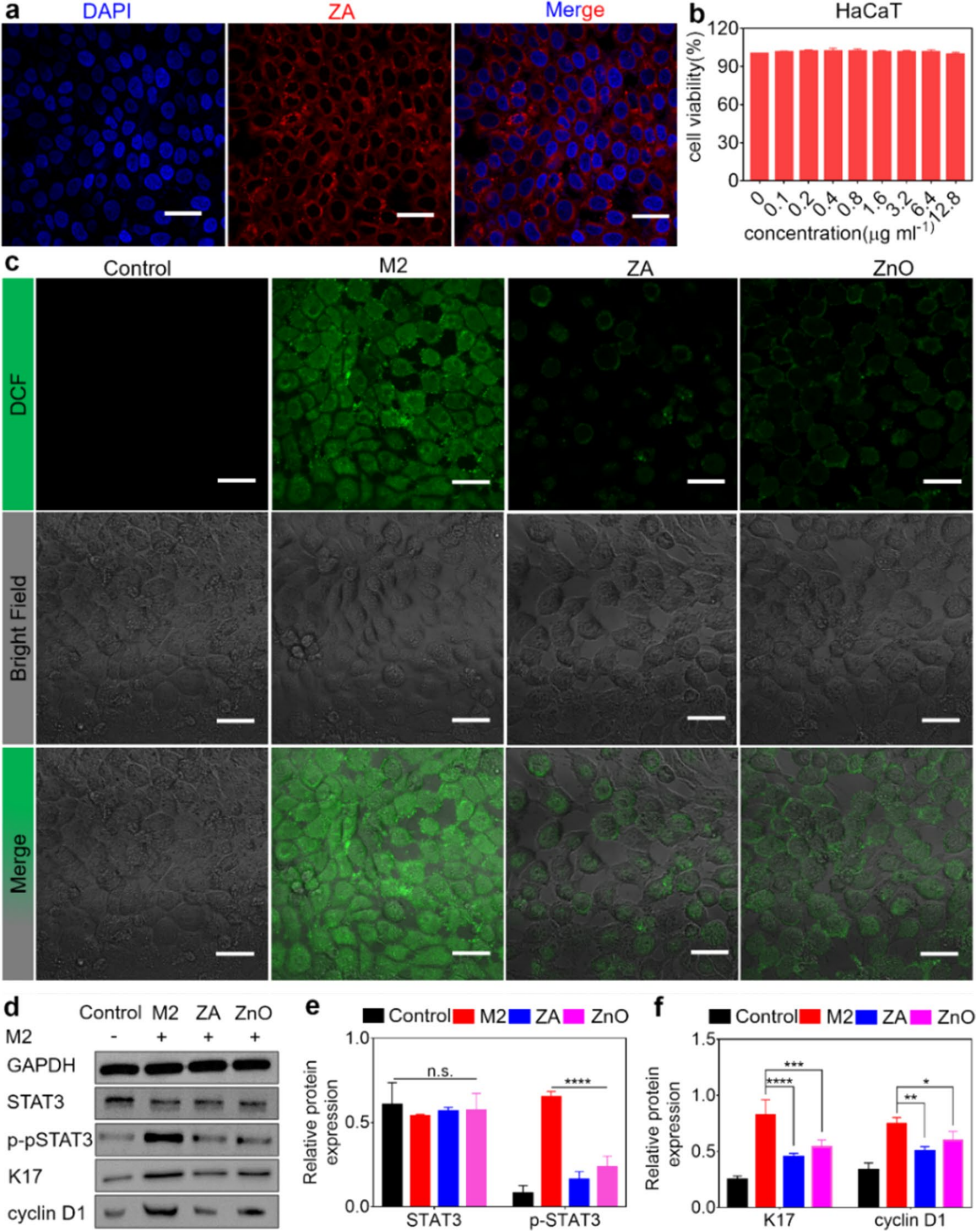
ZA inherent ablation of excessive ROS in KCs caused resistance to cyclin D1 expression and blocked self-amplifying K17/cytokine autoimmune loop. **a** CLSM images of HaCaT cells cellular internalization of NR-labeled ZA. Scale bar: 50 µm. **b** HaCaT cells viability after incubation with various concentrations of ZA determined by CCK-8 assays (n = 8, mean ± SEM). **c** CLSM images of intracellular ROS levels (green) induced in HaCaT cells by M2 with DCFH-DA probes. Scale bar: 50 μm. **d**–**f** Quantitative analysis of the activation of phospho-STAT3 and its downstream genes in M2-treated HaCaT cells by western blotting (n = 3, mean ± SEM). n.s. (not significant), ^***^p < 0.001, ^****^p < 0.0001vs. M2-treated group, M2: TNF-α and IL-17A, ZA: 0.1 wt% ZnO/Ag

Herein, a psoriasiform KCs model was employed in vitro to validate our speculation. As shown in Fig. 3a, b, ZA could be effectively assimilated in HaCaT cells and exhibited favorable cell biocompatibility. The combination of TNF-α and IL-17 (termed as M2), was used to stimulate HaCaT cells as a psoriasiform KCs model [35]. M2-evoked intracellular ROS production was detected using a standard fluorescent probe, 2′,7′-dichlorofluorescein diacetate (DCFH-DA). Compared with HaCaT cells challenged by M2, both the ZA- and ZnO-pretreated groups exhibited low intensity green fluorescence, implying that they had comparable effects on obliterating excessive ROS in M2-stimulated KCs (Fig. 3c). The generation of cellular ROS has been contributed to the mitochondrial malfunction, which has a relevant link with chronic inflammatory diseases [36, 37]. Various stress conditions, including increased metabolic rates, hypoxia, or membrane damage, markedly induce mitochondrial ROS production. As the transformation in the redox potential of the mitochondrial membrane is representative of mitochondrial dysfunction, resulting in ROS generation. Therefore, JC-1 staining was employed to determine the disruption of the mitochondrial membrane. In comparison with the M2-treated group, Confocal images showed a lower intensity green fluorescence appeared in the ZA and ZnO treatment group, suggesting the similarly protective effect of ZA and ZnO on mitochondrial membrane potential and the inhibition of ROS generation upon TNF-α stimulation (Additional file 1: Fig. S14). On account of these results, we deeply explored the potential molecular mechanism of the ZA. As anticipated, the western blotting analysis demonstrated that the ZA treatment group remarkably decreased the phosphorylation of STAT3 compared with the M2-treated group, accompanied by the reduction of the downstream gene expression of K17 and cyclin D1. While the ZnO-treated group showed an inferior effect to ZA treatment group (Fig. 3d–f). In parallel, the qRT–PCR data showed that ZA treatment significantly downregulated chemokines, including CXCL1, CCL20 and IL-1β (Additional file 1: Fig. S15). Therefore, our hypothesis was demonstrated by the abovementioned data.

### The decoration of nanomicelles affected the composite hydrogel penetration in psoriatic skin

**Fig. 4:**
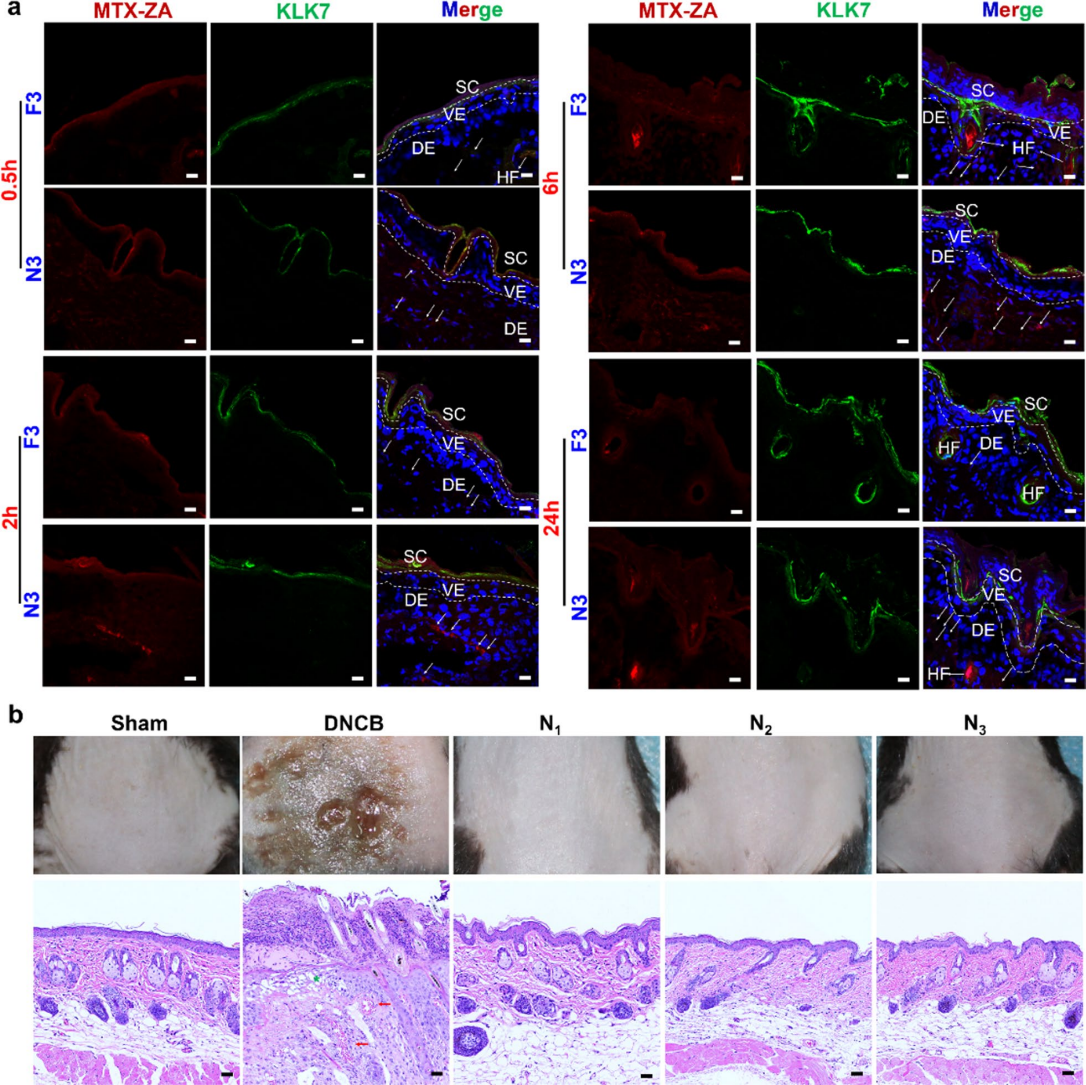
Skin permeability and irritation assessments of various formula hydrogels. **a** Penetration of NR-labeled MTX-ZA (red) through psoriatic skin at 0.5, 2, 6, and 24 h after administration was observed by CLSM. **b** Photograph and histopathological images of mice back skin in skin irritation study (the green asterisk: confluent nests of apoptotic keratinocytes, the red arrows: perivascular infiltrate of lymphocytes). Scale bars:50 μm, the nuclei were stained with DAPI (blue); KLK7 (green) was dyed to distinguish the SC layer, HF (hair follicles), SC (stratum corneum), VE (visible epidermal), DE (dermis). The white arrows indicate NR-labeled MTX-ZA. Sham: control group, DNCB: 1% 2,4-dinitrochlorobenzene (DNCB)-induced contact dermatitis groups, F_3_: Car@MTX-ZA hydrogel, N_1_: Car@NMs@MTX hydrogel, N_2_: Car@NMs@ZA hydrogel, N_3_: Car@NMs@MTX-ZA hydrogel

Inspired by the results of experiments in vitro, the Polycaprolactone-Polyethyleneglycol-Polycaprolactone (PCL-PEG-PCL)-based nanomicelles were engineered to endow MTX-ZA with promoted ability in transdermal permeation, and then they were embedded into the hydrogel formulation to suit for external skin application. Before exploring the therapeutic effects of different hydrogels, we firstly examined the skin penetration and irritation of our composite hydrogels in mice model. To compare the skin permeability performance of the Car@MTX-ZA hydrogel with that of the Car@NMs@MTX-ZA hydrogel, we analyzed the biodistribution and accumulation of MTX-ZA in different cutaneous layers of the psoriatic lesion using CLSM. As shown in Fig. 4a, confocal images revealed that after 0.5 h of treatment, the red fluorescence of the N_3_ group was found under SC, but the intensity of F_3_ was negligible. After 2 h and 6 h of topical administration, N_3_ displayed a higher fluorescent signal in the dermis than F_3_ in a time-dependent manner. After 24 h, the deposition of MTX-ZA in N_3_ remained slight in the dermis but was undetectable in F_3_, indicating that a particle size of 200 nm could permeate the epidermis [38], and the existence of nanomicelles had provided them with enhanced penetration depth and prolonged residence duration [13]. Notably, it is well known that particles with diameters of approximately few hundred nanometers can be effectively deliver into skin via skin appendages [39]. Consistency with Fig. 4a, a higher red fluorescence in hair follicles was observed. Altogether, these accumulation findings illustrated that the Car@NMs@MTX-ZA hydrogel efficiently penetrated the SC, even with a thickened epidermis in psoriatic lesions. Considering that topical MTX and metal nanoparticles might cause skin irritation and allergenic dermatitis [40, 41], a skin irritation study was performed to estimate the sensitization of these hydrogels. The irritation scores were the highest in the 1% 2,4-dinitrochlorobenzene (DNCB)-induced contact dermatitis groups (Fig. 4b). The F_1_ group showed slight scaly symptoms, and the other groups were negligible (Additional file 1: Figs. S16 and S17). Corresponding skin histopathological examination of these various groups was also performed, and the results were presented in Fig. 4b and Additional file 1: Fig. S17. The DNCB group revealed that confluent nests of apoptotic keratinocytes, epidermal regenerative changes and moderate perivascular infiltrate of lymphocytes in the superficial dermis. Minor proliferation of keratinocytes and subtle infiltration of leukomonocytes in the dermis were shown in F_1_ (Additional file 1: Fig. S17), and the remaining groups were without histopathological changes. Collectively, these results illustrated that our composite hydrogels had satisfactory biocompatibility and were hypoallergenic for skin application.

### Efficacy of the composite hydrogel transdermal therapy on IMQ-induced psoriasiform skin inflammation

**Fig. 5:**
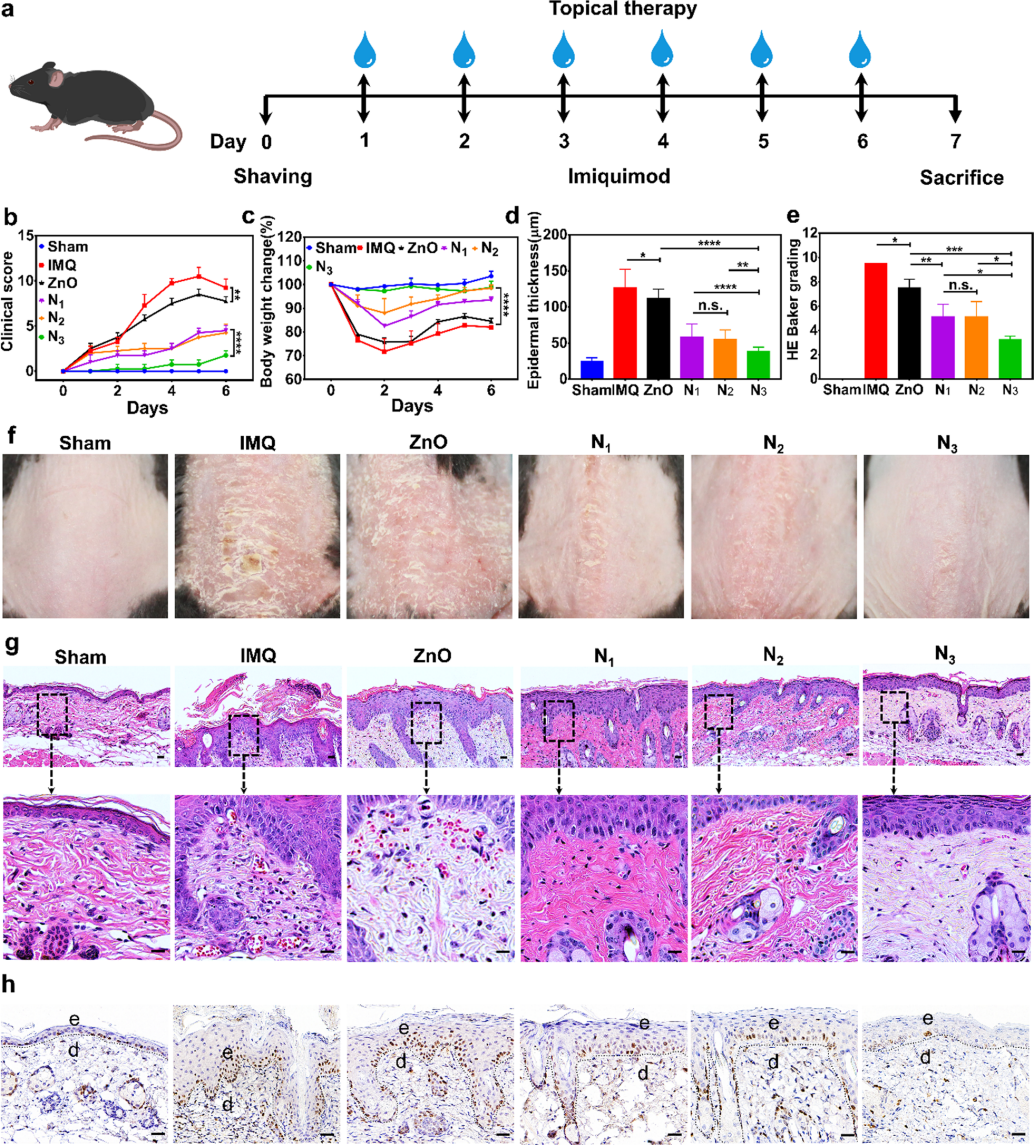
Transdermal therapy efficacy of various formula hydrogels on IMQ-induced psoriasiform skin inflammation (therapeutic dosage: 0.5 mL/daily). **a** Experimental scheme of antipsoriatic treatment. **b** PASI scores of skin lesions were monitored. **c** Change of body weight in percentage. **d** Quantification of the epidermal thickness. **e** HE Baker grading of the dorsal skin sections stained with H&E. **f** Representative images of the dorsal skin. **g** Representative H&E images of the dorsal skin sections. **h** Representative immunohistochemistry images of KI67^+^ cells in the dorsal skin sections. n = 4, mean ± SEM, scale bars:20 μm, n.s. (not significant), ^*^p < 0.05, ^***^p < 0.001, ^****^p < 0.0001 vs. IMQ group, Sham: control group, IMQ: imiquimod group, N_1_: Car@NMs@MTX hydrogel, N_2_: Car@NMs@ZA hydrogel, N_3_: Car@NMs@MTX-ZA hydrogel

The above findings suggested that the Car@NMs@MTX-ZA hydrogel with a self-therapeutic efficacy could better alleviate proinflammatory effects and relieve psoriasis symptoms. Hence, we established an IMQ-induced psoriatic mouse model to examine the effects of Car@NMs@MTX-ZA hydrogel in vivo [42]. As shown in Fig. 5a, topical administration of 5% imiquimod cream and concurrent application of different formula hydrogels were conducted with daily doses for six consecutive days, while the model group (IMQ) was managed with blank Carbopol hydrogel. Among these various treatments, N_3_ exhibited the most remarkable reduction of epidermal thickness, PASI scores and HE Baker grading (acanthosis and Munro microabscesses in epidermis, angiotelectasis and mononuclear cells infiltration in dermis), while the mouse weight remained constant, presenting superior efficacy to other groups (Fig. 5b–g and Additional file 1: Table S4). Likewise, the spleen length and spleen weight index, indicators of systemic immune responses, were also significantly reduced in N_3_ (Additional file 1: Fig. S18a, b). A remarkable decrease in the number of KI67^+^ KCs, a cell proliferation marker, was observed in the N_3_ (Fig. 5h and Additional file 1: Fig. S18c). An equivalent amount of free MTX was also loaded on N_1_, but it did not appear to be nearly as effective as N_3_, which was attributed to the intrinsic anti-inflammation of ZA. In comparison with other groups, ZnO only showed minor downregulation of all the evaluation indexes. As depicted in Fig. 5b-g and Additional file 1: Fig. S19a-h, for all groups not coated by PCL-PEG-PCL (F_1_-F_3_), their therapeutic effects were not matched for the PCL-PEG-PCL decorated groups (N_1_-N_3_), in terms of all the evaluation metrics. What's more, MTX and ZA alone presented almost the same therapeutic effects, ascertaining that ZA could be identified as a sort of self-therapeutic nanoparticle without side events of MTX. Taken together, this Car@NMs@MTX-ZA hydrogel was conferred with good comprehensive properties, especially optimized noninvasive transdermal drug delivery performance and augmentation of topical MTX therapeutic effects.

### The composite hydrogel ameliorated multiple psoriatic cytokines and the infiltration of proinflammatory macrophages in lesion skin

**Fig. 6:**
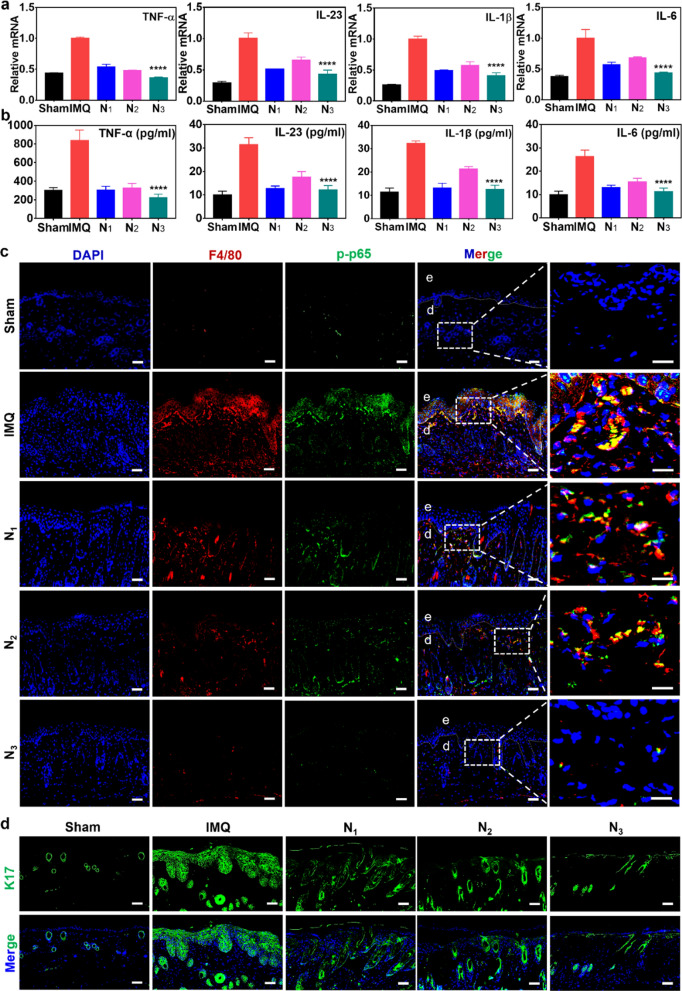
Impediment of IMQ-induced inflammatory circuits caused by Car@NMs@MTX-ZA hydrogel. **a**, **b** qRT–PCR and ELISA analysis of innate cytokine profiles in dorsal skin lesions after different treatments (n = 4, mean ± SEM). **c** Immunofluorescence co-staining of F4/80 (green) and p-p65 (red) in the dorsal skin lesions, scale bars:20 μm. **d** Skin lesions were stained with K17 (green). Scale bars:20 μm, ^*^p < 0.05, ^***^p < 0.001, ^****^p < 0.0001 vs. IMQ group, sham: control group, IMQ: imiquimod group, N_1_: Car@NMs@MTX hydrogel, N_2_: Car@NMs@ZA hydrogel, N_3_: Car@NMs@MTX-ZA hydrogel

To further understand the mechanisms of different hydrogels, the psoriatic cytokines in lesioned skin were measured by qRT–PCR and ELISA analysis to evaluate the ameliorative effects of the Car@NMs@MTX-ZA hydrogel. The results showed that N_3_ prominently suppressed the mRNA and protein expressions of TNF-α, IL-23, IL-1β and IL-6 compared with the other groups (Fig. 6a, b). Next, we prepared to detect infiltrating proinflammatory macrophages via co-stained with F4/80 (the macrophage surface marker) and p-p65. Immunofluorescence assay showed that N_1_ and N_2_ downregulated phosphorylation of p65 in proinflammatory macrophages, further confirming the anti-inflammatory ability of ZA, but their effects were not as good as those of N_3_ (Fig. 6c). Thus, all these data validated that ZA possessed an immunomodulatory function, which could fortify the therapeutic effect of extrinsic MTX. Encouraged by the results of the biological activity of ZA in vitro, whether ZA could block K17 immune loop in activated KCs was further examined in vivo. Immunofluorescence analysis showed that the positive fluorescence intensity of K17 in N_3_ was fiercely reduced compared with that in N_1_ and N_2_. Meanwhile, N_1_ and N_2_ displayed comparable downregulation. These results suggested a significant blockade effect of N_3_ in the IMQ-induced psoriatic skin inflammatory circuit (Fig. 6d). In addition, the predominant biocompatibility of nanomaterials in vivo is a precondition for their biomedical applications. Therefore, the toxicology of the Car@NMs@MTX-ZA hydrogel was systematically studied by collecting skin, serum and major internal organs. As data presented in Additional file 1: Figs. S20a–d and S21a–d, after six days of treatment with these hydrogels, no apparent pathological changes were observed, regardless of the main organs, blood chemistry or hematology, indicating that these hydrogels only caused ignorable biotoxicity at the therapeutic dosage in vivo. Moreover, Ag^+^ content was measured in skin tissue and the main organs at days 1, 7, and 28 after the final local treatment. The Ag^+^ was mainly distributed in skin tissue, while lower Ag content accumulated in internal organs. At day 7, Ag^+^ was almost excreted from the skin and each organ (Additional file 1: Fig. S22).

## Conclusion

In this work, a multifunctional composite hydrogel based on ZnO/Ag nanoparticles with inherent self-therapeutic ability for psoriasis was constructed. In vitro experiments showed that ZnO/Ag nanoparticles inhibited the activation of p65 in proinflammatory macrophages and suppressed ROS-induced STAT3-cyclin D1 signaling in KCs, resulting in simultaneous blockade of the key nodes of innate and adaptive cytokine networks. In addition, ZnO/Ag could also serve as a nano-delivery platform to optimize the sustainable release of MTX. Meanwhile, the introduction of nanomicelles was conducive to the enhancement of transdermal delivery of nanocomposites through the thickened skin layers of psoriasis area. As a result, this hydrogel potentiated the immunomodulatory function of MTX in a psoriasis-like mouse model. In particular, the mulfunctional hydrogel is comprised of ZnO/Ag with extreme low concentration, which shows good biocompatibility. Therefore, this multifunctional hydrogel is believed to be a promising nanoplatform for the transdermal delivery of MTX, characterized by remarkable antipsoriatic efficacy and the potential for clinical translation.

## Materials and methods

### Materials and sample characterizations

Tin (II) 2-ethylhexanoate (92.5–100%), Polyethylene glycol, ε-Caprolactone (97%), Lipopolysaccharide (LPS), Carbopol 934, 2,4 Dinitrochlorobenzene (DNCB), Eucalyptus oil (92.5–100%), and phorbol 12-myristate 13-acetate (PMA) were purchased from Sigma Aldrich. Triethanolamine and Methotrexate were obtained from Macklin. 5% Imiquimod (IMQ) cream was purchased from Sichuan Mingxin Pharmaceuticals. Cell Counting-Kit 8 assay (CCK-8), Reactive Oxygen Species Assay Kit and T-AOC Assay Kit were purchased from Beyotime. Penicillin − streptomycin, trypsin, phosphate-buffered saline (PBS), DMEM and RMPI medium 1640 were purchased from Invitrogen. Fetal bovine serum (FBS) was obtained from Wisent Corporation. Cytokines IFN-γ, IL-23, TNF-α and IL-17A were obtained from PeproTech. STAT3, p-STAT3, Cytokeratin 17 and Kallikrein 7 (KLK7) antibodies were obtained from Abcam. p65 and p-p65 antibodies were obtained from Santa Cruz. F4/80 and CyclinD1 antibodies were purchased from Cell Signaling. Primers for qRT-PCR analysis were synthesized by Sangon Biotech. The morphologies, fluorescence, and chemical constitutions of the samples were determined by conventional methods according to our previous protocol or instruments [43]. Fourier transform infrared spectroscopy (FTIR) was examined by a Thermo Nicolet iN10, ^1^H NMR was recorded on a Bruker-400 MHz spectrometer, viscosity and storage/loss modulus (G′/G″) were documented using Kinexus Rheometer (Malvern Instrument, UK).

### Synthesis of ZnO/Ag and NMs@MTX-ZA nanoparticles

Firstly, AA-[Zn(OH)_4_]^2−^ (denoted as ZnO) were synthesized according to our previous research with some modification [20]. Briefly, 0.376 mmol of AA and 1.96 mmol of CTAB were dissolved using 150 mL deionized water and added into a 250 mL round-bottom flask. Next, 0.398 mmol of HMTA and 0.398 mmol of Zn(NO_3_)^2^∙6H_2_O were added into the mixed solution with stirred for 30 min, then the mixed solution was heated at 85 °C for 10 h to obtain the suspension, which was washed several times using absolute ethanol and deionized water, and then dried. Subsequently, three types of ZnO/Ag (ZA) nanoparticles (0.01%, 0.1% and 1 wt%ZA) containing different Ag amount were prepared via ZnO hollow microspheres laden with Ag nanoparticles by adjusting the concentration of AgNO_3_ according to our previous study [22]. Nanomicelles (PCL-PEG-PCL) and PCL-PEG-PCL decorated MTX-ZA (termed as NMs@MTX-ZA) was synthesized by previously reported method with some modification [13]. Briefly, 3.74 mL of PCL, 4.05 g stannous octoate and 2 g of PEG were heated and stirred at 140 °C for 24 h. After 24 h, the obtained copolymer was precipitated in icy distilled water, washed three times and lyophilized. Then, 50 mg of the obtained PCL-PEG-PCL, 10 mg of MTX and 10 mg of ZA were dissolved in 0.5 mL DMSO with stirred for 6 h. The mixed organic phase was added into 10 mL deionized water with continuous ultrasound for 30 min, then the obtained NMs@MTX-ZA was centrifugated at 10,000 rcf for 25 min. The a centrifugated deposit was resuspended in deionized water and lyophilized. Likewise, NMs@MTX and NMs@ZA were synthesized using the same protocol with the MTX or ZA alone.

### Evaluation of antioxidant activity

Total antioxidant capacity of 0.01 wt% ZA, 0.1 wt% ZA, 1 wt% ZA and ZnO were tested by ABTS rapid method using the T-AOC Assay Kit referring to the instructions.

### Preparation of Car@NMs@MTX-ZA hydrogel

Car@NMs@MTX-ZA, Car@NMs@MTX and Car@NMs@ZA hydrogels were obtained by loading NMs@MTX-ZA, NMs@MTX and NMs@ZA into hydrogels according to a previous report, repectivley [13]. Eucalyptus oil used as penetration enhancer was introduced into all hydrogels. Car@MTX, Car@ZA and Car@MTX-ZA were prepared in the same manner without the decoration of NMs.

### In vitro MTX release from Car@NMs@MTX-ZA hydrogel

MTX release profiles were performed in 20 mL phosphate buffer pH 7.4 at 37 °C with shaking at 120 rpm, respectively. 1 mL of samples loaded into dialysis membrane (3500 Da), respectively. The dialysis membrane was put into 20 mL of release media. At preset time intervals (2, 4, 8,12, 24, 48, 96, 144 h), 2 mL of release solution was collected, and substituted with an equal amount of fresh medium to maintain unchanged total volume. The collected release mediums were subjected to UV–vis spectrophotometer to analyze MTX concentration, the λ_max_ of MTX was 302 nm.

### Cell lines

HaCaT, Murine macrophage RAW264.7 and human monocytic leukemia THP-1 cells were purchased from the China Center for Type Culture Collection. HaCaT and RAW264.7 cells were grown in DMEM medium; THP-1 cells were cultivated in RPMI 1640 medium, All the culture medium complemented with 10% FBS, 1% penicillin and streptomycin. All cells were not contaminated with mycoplasma. Induction of THP-1 derived inflammatory macrophages: THP-1 cells were cultured with 100 ng/mL PMA in 6-wells cell culture plates for 24 h to obtain differentiated and plastic-adherent M0 macrophages, the culture medium of M0 cells was replaced with fresh RPMI 1640 media to obtain resting macrophages and then challenged by 100 ng/mL LPS plus 20 ng /mL IFN-γ to generate inflammatory macrophages (M1 cells). Establishment of in vitro psoriatic model: HaCaT cells were co-stimulated with 10 ng/mL TNF-α and 200 ng/mL IL17A (denoted as M2) in DMEM for 24 h to recapitulate features of psoriasis [35]. The control cells used in all measurements were exposed with the culture medium containing phosphate buffered saline in similar amount as other treatments.

### In vitro cellular uptake and cell viability assays

THP-1cells and HaCaT cells were incubated with Nile Red (NR)-labeled ZA NPs for 4 h, followed by the red fluorescence images were assessed by CLSM (LMS-800, Carl Zeiss) to confirm cellular uptake of ZA NPs. THP-1cells, HaCaT cells and RAW264.7 cells were exposed with 0.01 wt% ZA, 0.1% wt ZA and 1% wt ZA for 24 h, followed by evaluating cell viability using CCK-8 according to the kit introductions.

### In vitro ROS assay and JC-1 staining

Intracellular ROS generation and the potential of the mitochondria membranes were estimated by DCFH-DA and JC-1 staining respectively. Briefly, HaCaT cells (2–3 × 10^5^ cells/mL) were cultured into 1 mL culture medium of confocal plates. On the second day, the cells were pretreated with fresh DMEM medium containing 0.2 μg/mL 0.1 wt% ZA and 0.2 μg/mL ZnO for 18 h, respectively. then the cells were stimulated by another 6 h under TNF-α and IL-17A stimulation. Finally, the cells were cultured with DCFH-DA for 30 min at 37 °C in the dark to determine ROS level. After 30 min, the cells were washed using phosphate buffered saline. Oxidative stress of HaCaT cells was captured by using CLSM. At the same time, the potential of the mitochondria membranes was inspected by mitochondrial membrane potential assay kit with JC-1 according the kit introductions under the same experimental conditions.

### qRT-PCR analysis

Total RNA from cells and mice tissues were withdrawned with TRIzol (Life technogies, 15,596,026) according to the kits' instructions. Genomic DNA was eliminated and cDNA was then reverse transcribed with the total RNA (1 µg), a primeScript RT reagent kit (TaKaRa, RR047A) was applied to reverse transcription to synthesize cDNA. The obtained cDNA (1μL) was received to qRT-PCR analysis using Novostart SYBR qPCR SuperMix Plus (Novoprotein, E096-01B). The results were normalized to ACTB, and the 2^−ΔΔCt^ method was performed to quantify. All primers were obtained from Sangon Biotech Co., Ltd and their sequences were listed in Additional file 1: Table S2.

### Western blot

Samples obtained from cells were lysed, separated by electrophoresis and transferred to polyvinylidene fluoride (PVDF) membranes (MerckMinipore, IPVH00010). The proteins were incubated with the primary antibodies: GAPDH (ZSGB-BIO, TA-08, 1:10000 dilution), p-STAT3 (Abcam, Ab267373,1:2000 dilution), STAT3 (Santa Cruz, sc-8019, 1:1000 dilution), p65 (Abcam, bs-0466R, 1:1000 dilution), p-p65 (Santa Cruz, sc-136548, 1:1000 dilution), Cytokeratin 17 (Abcam, Ab51056, 1:10,000 dilution), CyclinD1 (Cell Signaling Technology, 55506 T, 1:1000 dilution). Goat anti-rabbit or goat anti-mouse antibodies (ZSGB-BIO, ZA-2305 or ZA-2301, 1:10,000 dilution) was used to label the primary antibodies and further probed using ECL reagents (Thermo). The band intensities of the images were quantified using ImageJ.

### ELISA analysis

Supernatants of cell culture and tissue homogenate were collected. The TNF-α, IL-23, IL-6 and IL-1β levels were measured using the TNF-α Detection Kit (JYM0110Hu, GR2021-03), IL-23 ELISA kit (JYM0083Hu, 20,210,810), IL-6 ELISA kit (JYM0140Hu, GR2021-03), IL-1β ELISA kit (JYM0083Hu, GR2021-03) according to the manufacturer's protocol.

### In vivo skin irritation

Skin irritation assay was performed according to previous research with some modifications [44]. Briefly, Mice were randomized into 8 groups (3 mice in each group): control group with acetone, irritant dermatitis group with 1% 2,4-dinitrochlorobenzene (DNCB) in acetone, other groups were treated daily (0.5 mL) with Car@NMs@MTX-ZA hydrogel, Car@NMs@ZA hydrogel, Car@NMs@MTX hydrogel, Car@MTX-ZA hydrogel, Car@ZA hydrogel, and Car@MTX hydrogel on the back of mice for six days, respectively. The skin potential irritation of these hydrogels was compared with the irritant dermatitis group. The control group did not manage with any treatment. Signs of skin irritation of mice were monitored every day before and after hydrogels treatment throughout the period. An overall irritation score ranging from 0 to 4: 0 = no difference, 1 = mild erythema, 2 = well defined erythema, 3 = strong erythema, 4 = very strong erythema. Histopathological analyses of the dorsal skin of mice were further employed to confirm the skin irritation profiles.

### In vivo skin penetration of Car@NMs@MTX-ZA hydrogel in psoriasis

To evaluate the biodistribution and skin permeability of NMs@MTX-ZA in IMQ mice. On day 4, Nile red (NR)-labeled Car@NMs@MTX-ZA and Car@MTX-ZA hydrogels were applied to the dorsal skin of an IMQ-induced psoriatic mouse model (0.5 mL/2 × 3 cm^2^). The psoriatic skin tissues were collected and then embedded into O.C.T. compound at a time intervals of 0.5, 2, 6 and 24 h. Next, these tissues were frozen using liquid nitrogen and sliced at 7 μm by Cryotome, and then were fixed with 4% paraformaldehyde. Skin sections were stained with KLK7 to distinguish from epidermis and dermis, and nuclei were counterstained with DAPI. Finally, slides were mounted by antifade mountant. The distribution of MTX-ZA in psoriatic lesions was captured by CLSM.

### Animals and psoriasiform model

Six to eight-week-old female C57BL/6 J mice were purchased from Ji'Nan Pengyue Laboratory Animal Breeding Co., Ltd. All mice were fed and housed in specific pathogen-free conditions of the Anhui Medical University laboratory animal center. The Ethical Committee of Anhui Medical University approved all animal experiments. (Approved number: LLSC20210077). Psoriasiform skin inflammation was established by 5% Imiquimod cream, according to the literature [42]. Female mice with shaved back hair were randomly divided into 9 groups (n = 4). IMQ cream (62.5 mg) was applied daily to the shaved back area of each group except for the sham group. After 4 h, the experimental groups were treated with various hydrogels (therapeutic dosage: 0.5 mL/daily). All treatment groups were conducted for 6 consecutive days, and mice were weighed and monitored daily for PASI score (Psoriasis Area and Severity Index was employed to evaluate IMQ-induced erythema, scales, and thickness). On day 7, mice were sacrificed following injection with euthanasia solution followed by skin tissue and spleen dissociation. Samples from these mice were collected immediately, flash frozen, stored at − 80 °C for qRT–PCR and ELISA analysis, and placed into formalin for histological analysis.

### Histopathology, immunofluorescence and immunohistochemistry assays

Mouse dorsal skin tissues were collected and fixed with 4% formaldehyde for 24 h followed by dehydrated and embedded into paraffin. Next these tissues were sectioned at 7 μm and stained with H&E. Histopathology Slices were scanned using 3DHISTECH. For immunofluorescence and immunohistochemistry, 4% formaldehyde-fixed paraffin-embedded dorsal skin sections were deparaffinized, and stained with KI67, F4/80, p-p65, and K17. The images were recorded on an Olympus microscope (IX73). And the histologic Baker grading system for the psoriasiform model refers to Additional file 1: Table S3.

### In vivo toxicity estimation

To evaluate the accumulation of Ag^+^ in the skin and main internal organs, healthy C57BL/6 J mice were topically administrated with Car@NMs@MTX-ZA hydrogels daily as described above. They sacrificed at predetermined time points (the 1st,7th, and 28th day) post treatment. The skin tissue and the main organs were collected to measure the Ag^+^ content using ICP-OES. Another three healthy C57BL/6 J mice were used for the control. blood samples (∼1.0 mL) were collected for blood biochemistry test by using the standard kits according to the manufacturer's instructions. For histological assessment, every mouse was sacrificed and the main organs (heart, liver, spleen, lung, and kidney) were collected for hematoxylin and eosin staining.

### Statistical analysis

Statistical analysis was executed using a one-way ANOVA. The differences were considered to be statistically significant for a *p*-value (^*^p < 0.05, ^**^p < 0.01, ^***^p < 0.001, ^****^p < 0.0001).

## Supplementary Information

**Additional file 1. **The experimental section, SEM images, ^1^H-NMR spectra, UV-Vis-NIR absorption spectra, skin irritation assays, serum biochemistry analysis and biodistribution of Ag^+^ results after topical administration, and H&E staining results for major organs are provided in the supplementary information. The Additional Information is available free of charge on the Journal of Nanobiotechnology Publications website at http://jnanobiotechnology.biomedcentral.com.

## Data Availability

The datasets used and analyzed during the current study are available from the corresponding author on reasonable request.

## Abbreviations

TNF-α: Tumor necrosis factor alpha-like
IL-23: Interleukin 23
IL-6: Interleukin 6
IL-36R: Interleukin 1 receptor like 2
h-BD2: Defensin beta 4A
S100A7: S100 calcium binding protein A7
CXCL1: CXC chemokine ligand 1
IL-1β: Interleukin 1 beta
CCL20: CC chemokine ligand 20
K17: Keratin 17
