# Supplementary material for: A multifunctional composite hydrogel as an intrinsic and extrinsic coregulator for enhanced therapeutic efficacy for psoriasis
Source: J Nanobiotechnology. 2022 Mar 24;20:155. doi: 10.1186/s12951-022-01368-y (PMC8943972; doi:10.1186/s12951-022-01368-y)
Supplement: Supplementary file 1 — Additional file 1. The experimental section, SEM images, 1H-NMR spectra, UV-Vis-NIR absorption spectra, skin irritation assays, serum biochemistry analysis and biodistribution of Ag+ results after topical administration, and H&E staining results for major organs are provided in the supplementary information. The Additional Information is available free of charge on the Journal of Nanobiotechnology Publications website at http://jnanobiotechnology.biomedcentral.com. [file 12951_2022_1368_MOESM1_ESM.docx]

**Supplementary Information**

**A Multifunctional Composite Hydrogel as an Intrinsic and Extrinsic Coregulator for Enhanced Therapeutic Efficacy for Psoriasis**

Jiangmei Xu ^†, #^, Hao Chen^§, #^, Zhaoyou Chu^§^, Zhu Li^†^, Benjin Chen^§^, Jianan Sun^§^, Wei Lai ^‡^, Yan Ma^§^ , Yulong He^∇^, Haisheng Qian^§,^ **^*^**, Fei Wang^∇,^ **^*^**, Yunshen Xu^†,^ **^*^**

*^†^Department of Dermatovenerology, The Seventh Affiliated Hospital, Sun Yat-sen University, Shenzhen, Guangdong, P. R. China*

*^‡^Department of Dermatovenerology, The Third Affiliated Hospital, Sun Yat-sen University, Guangzhou, Guangdong, P. R. China*

*^§^School of Basic Medical Sciences, School of Biomedical Engineering, Research and Engineering Center of Biomedical Materials, Anhui Provincial Institute of Translational Medicine*

*^∇^Center for Digestive Disease, The Seventh Affiliated Hospital, Sun Yat-sen University, Shenzhen, Guangdong, P. R. China*

**Supplementary figures**


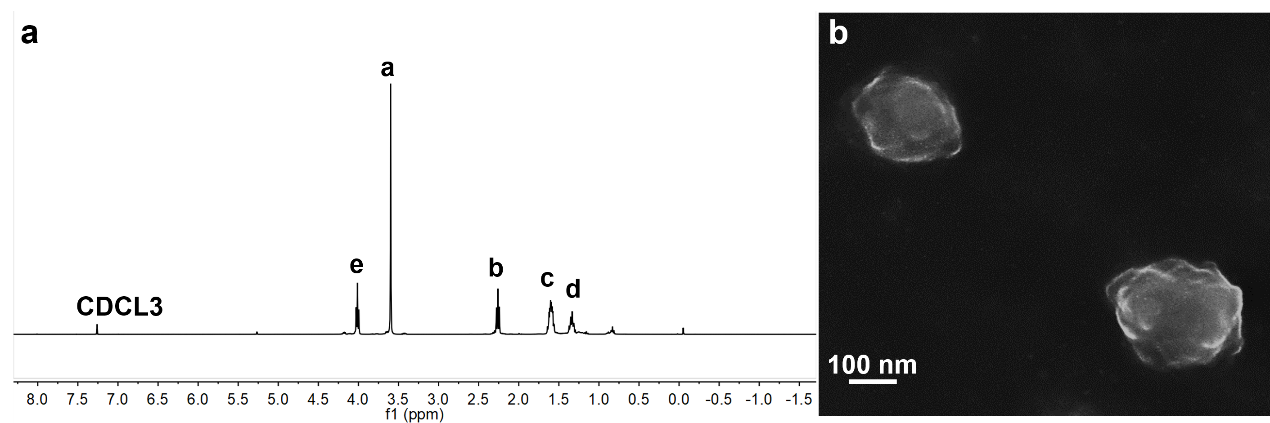


**Fig. S1.** Preparation and characterization of NMs@MTX-ZA. (a) Proton nuclear magnetic resonance spectra of PCL-PEG-PCL. (b) SEM image of NMs@MTX-ZA.

**
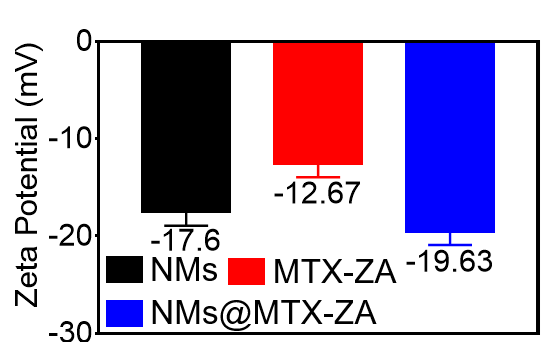
**

**Fig. S2.** Zeta potential analyses of the NMs, MTX and [NMs@MTX-ZA.](mailto:NMs@MTX-ZA.(b))


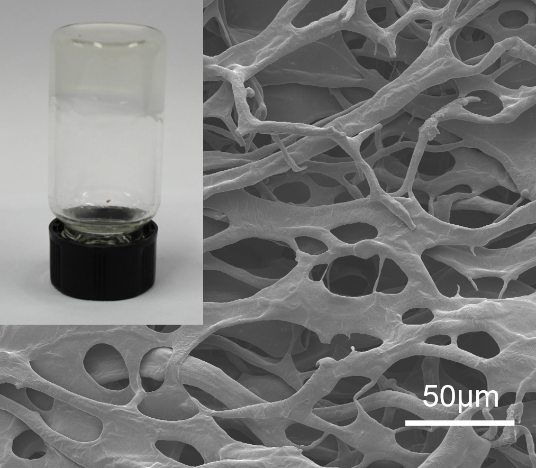


**Fig. S3.** SEM image of porous structures and digital picture of the Carbopol hydrogel


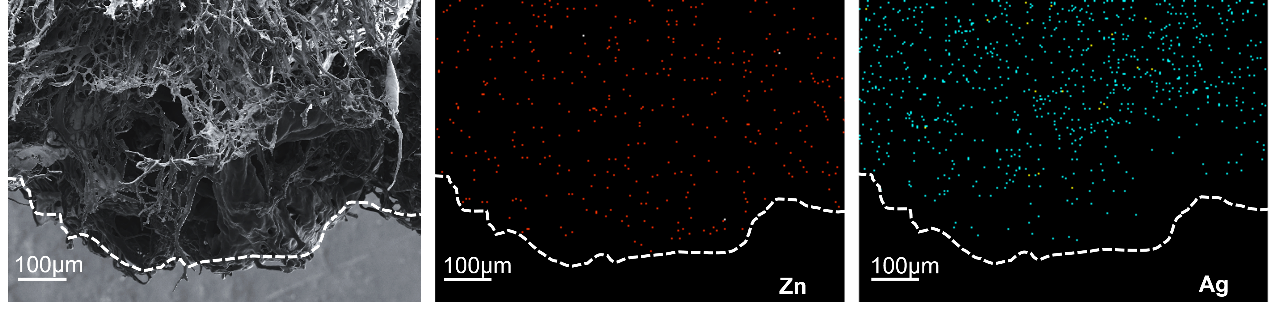


**Fig. S4.** The EDS mapping results of Zn and Ag elements in Car@NMs@MTX-ZA hydrogel.


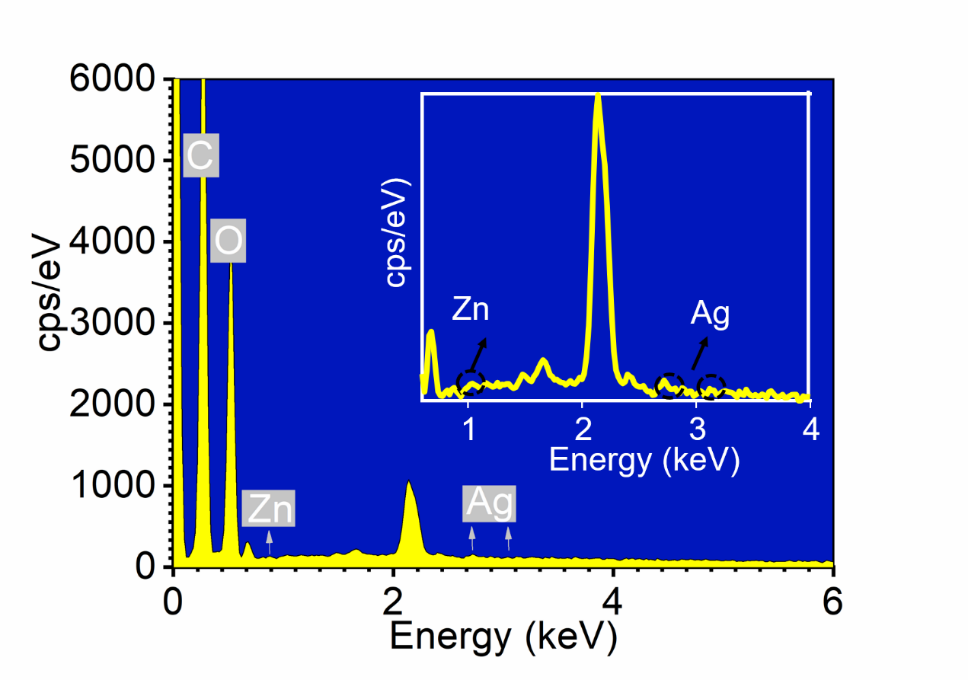


**Fig. S5.** Energy dispersive X-ray spectra of the as-prepared Car@NMs@MTX-ZA hydrogel.


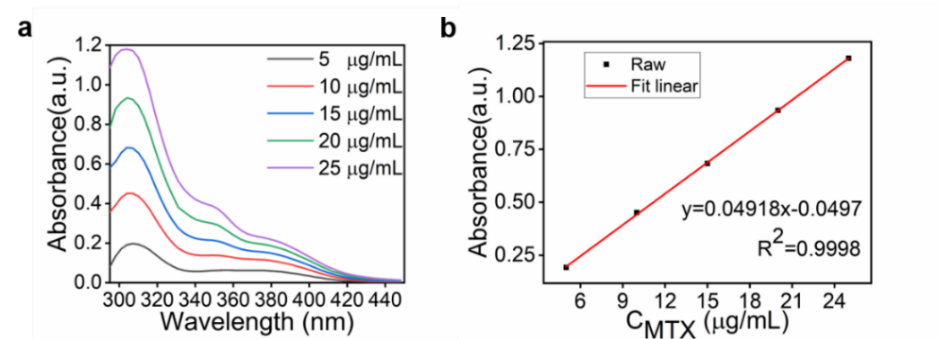


**Fig. S6.** (a-b) Absorbance of MTX at 302 nm in a concentration-dependent manner.

**
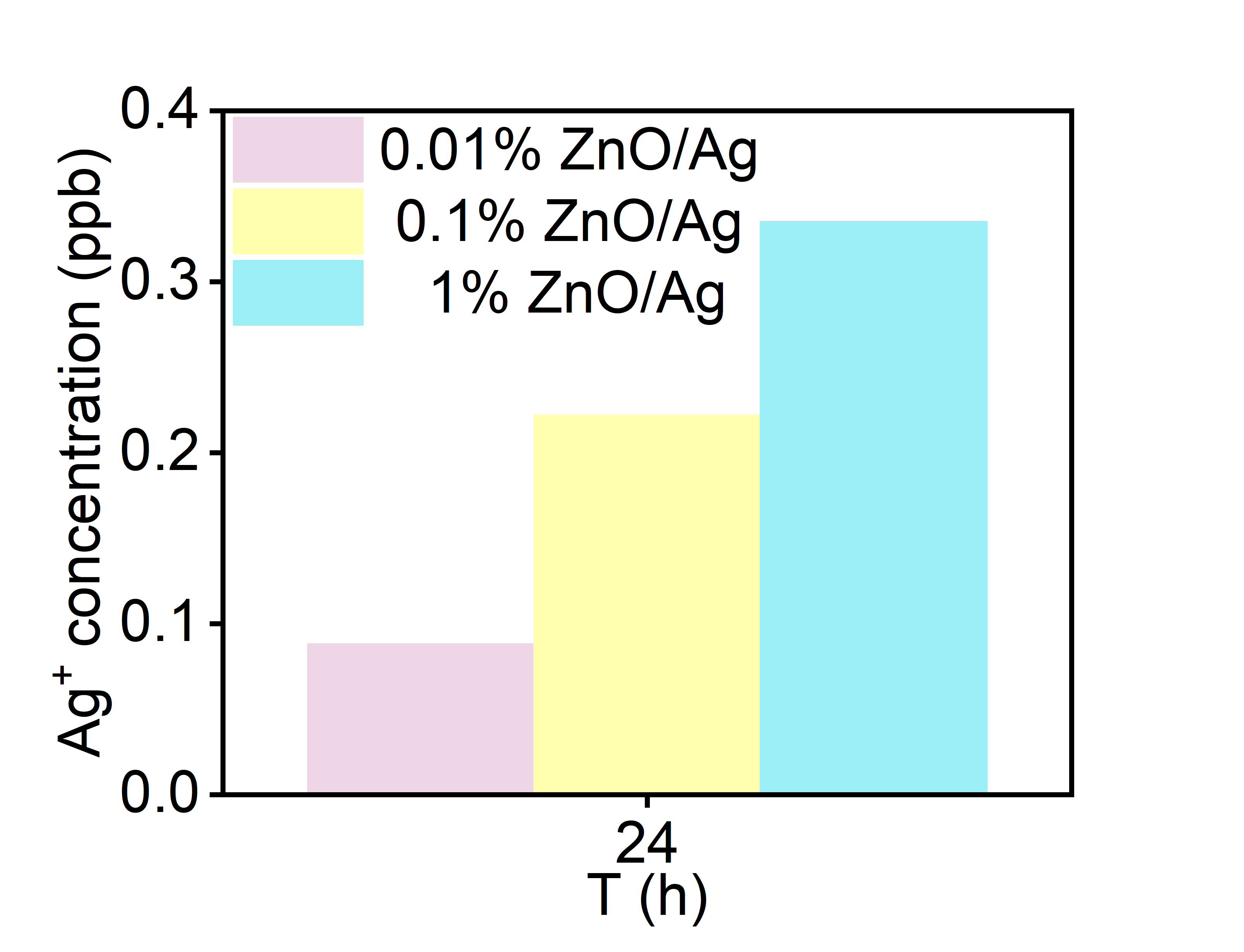
**

**Fig. S7.** Ag^+^ released from 0.01 wt% ZA, 0.1 wt% ZA and 1 wt% ZA at the time point of 24h in phosphate buffer solution (pH=7.4).


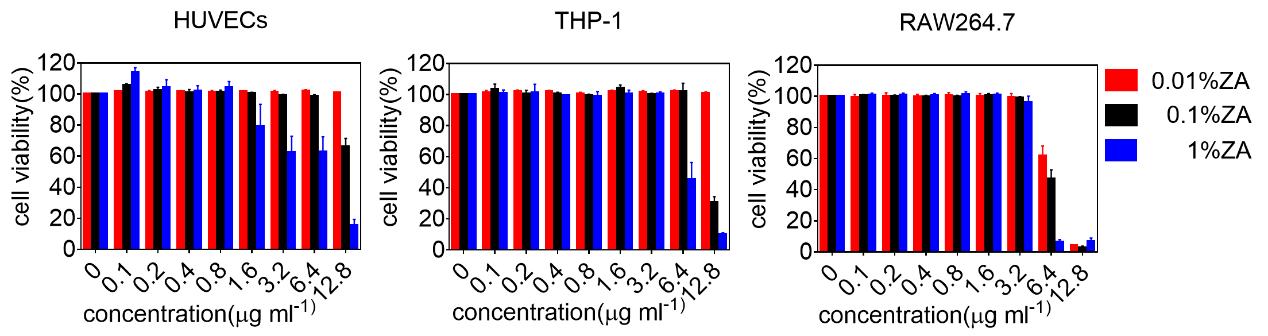


**Fig. S8.** Relative cell cytocompatibility analysis of HUVECs, THP-1 and RAW264.7 cell lines exposed with 0.01 wt%ZA, 0.1 wt% ZA and 1 wt% ZA (n = 8, mean ± SEM).


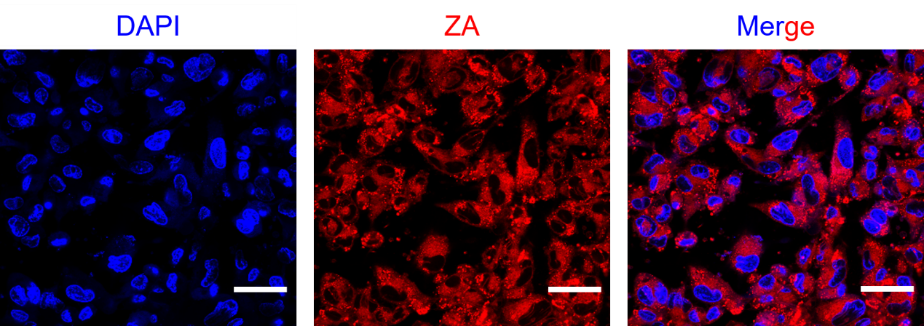


**Fig. S9.** CLSM images of LPS and IFN-γ treated THP-1 cells after 4h incubation with 0.1 wt% ZA. Scale bar: 50 μm.


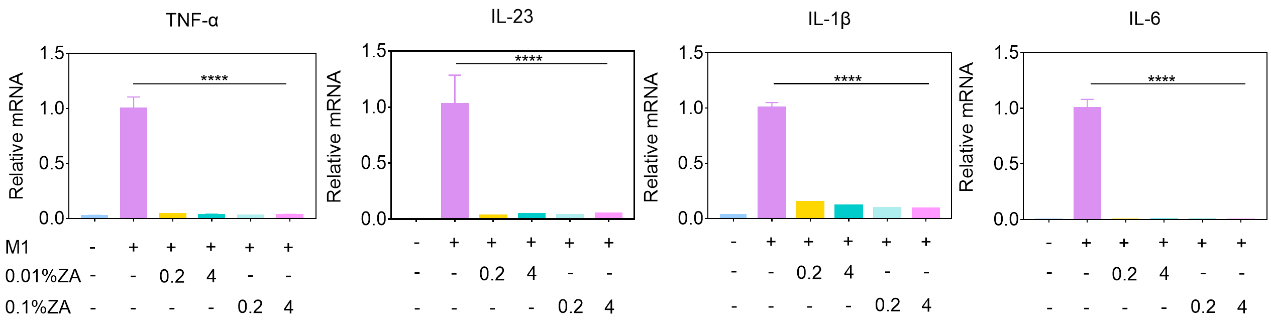


**Fig. S10.** mRNA levels of innate cytokine profiles M1 cells with or without treatment of 0.01 wt% ZA and 0.1 wt% ZA were measured by quantitative PCR. (n = 3, mean ± SEM), ^****^*p*<0.0001vs. M1-treated group, M1: LPS+IFN-γ, 0.01 wt% ZA (μg/mL), 0.1 wt% ZA (μg/mL).


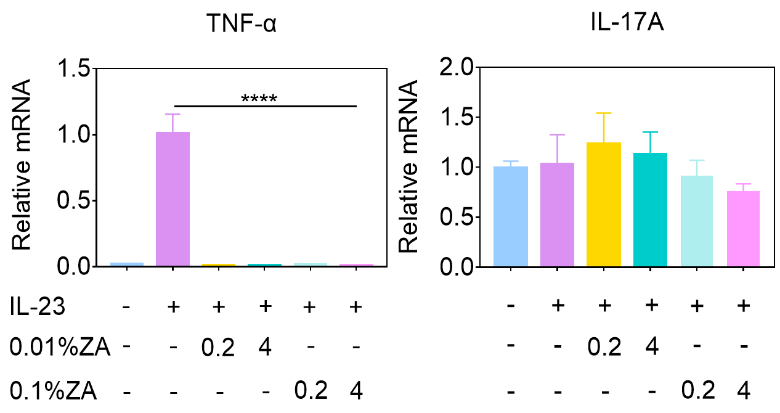


**Fig. S11.** mRNA levels of TNF-α and IL-17A from M0 cells in the presence of IL-23 stimulation with or without treatment of 0.01 wt% ZA and 0.1 wt% ZA were measured by quantitative PCR and ELISA. (n = 3, mean ± SEM), ^****^*p*<0.0001vs. IL-23-treated group, 0.01 wt% ZA (μg/mL), 0.1 wt% ZA (μg/mL).


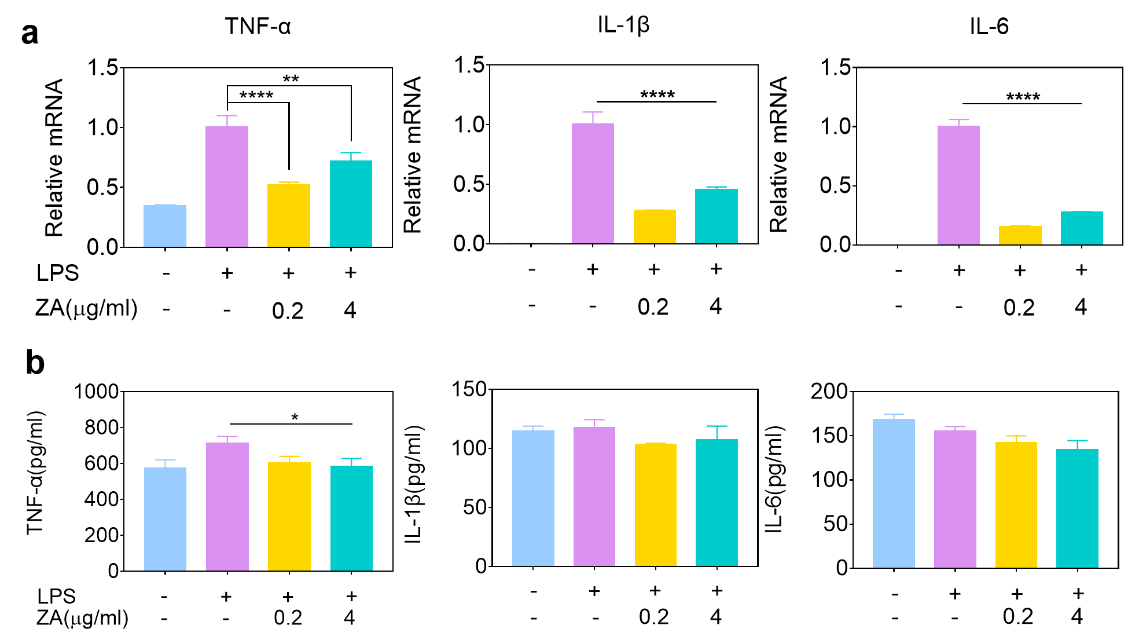


**Fig. S12.** mRNA levels of innate cytokine profiles from RAW264.7 cells in the presence of LPS stimulation with or without treatment of 0.1 wt% ZA were measured by quantitative PCR and ELISA. (n = 3, mean ± SEM), ^*^*p*<0.05, ^***^*p*<0.001, ^****^*p*<0.0001vs. LPS-treated group, ZA: 0.1% ZA.


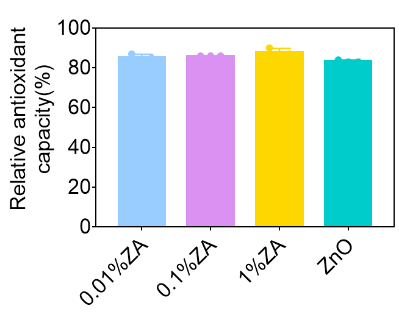


**Fig. S13.** The antioxidant capacity of 0.01 wt% ZA, 0.1 wt% ZA, 1 wt% ZA and ZnO.


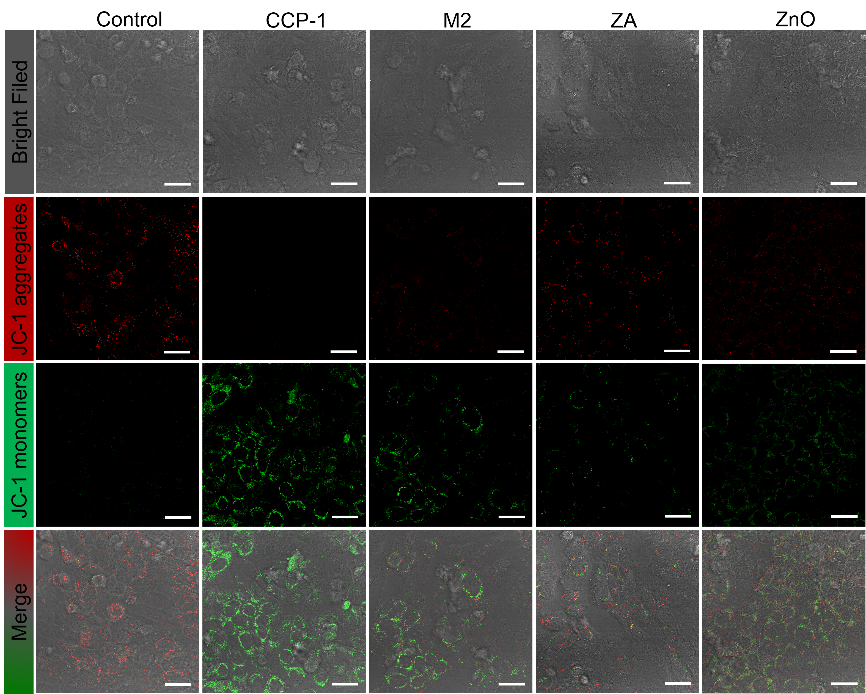


**Fig. S14.** The mitochondrial membrane potential was monitored by JC-1 staining. scale bars:50 μm


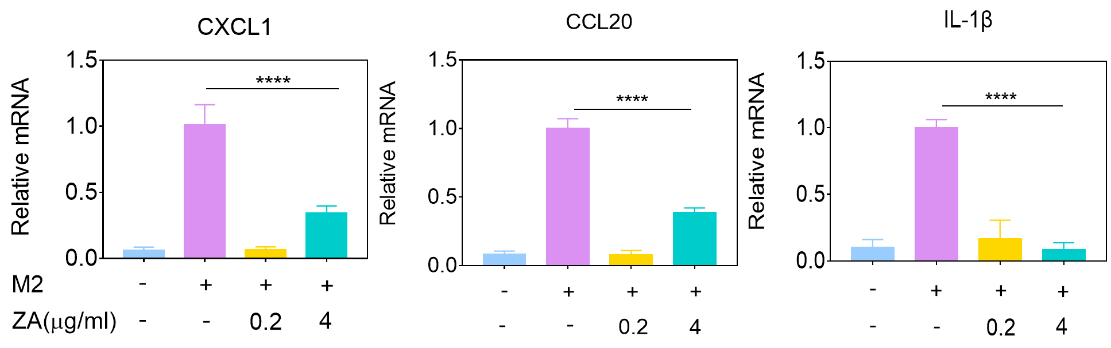


**Fig. S15.** mRNA levels of adaptive cytokine profiles from HaCaT cells in the presence of M2 stimulation with or without treatment of ZA were measured by quantitative PCR. (n = 3, mean ± SEM), ^*^*p*<0.05, ^***^*p*<0.001, ^****^*p*<0.0001vs. M2-treated group, M2: TNF-α and IL-17A, ZA: 0.1 wt% ZA.


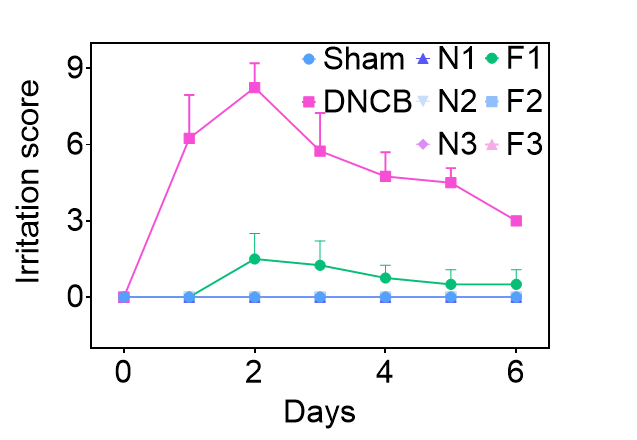


**Fig. S16.** Skin irritation after different formula hydrogels treatment was monitored by irritation score (n=4, mean ± SEM).


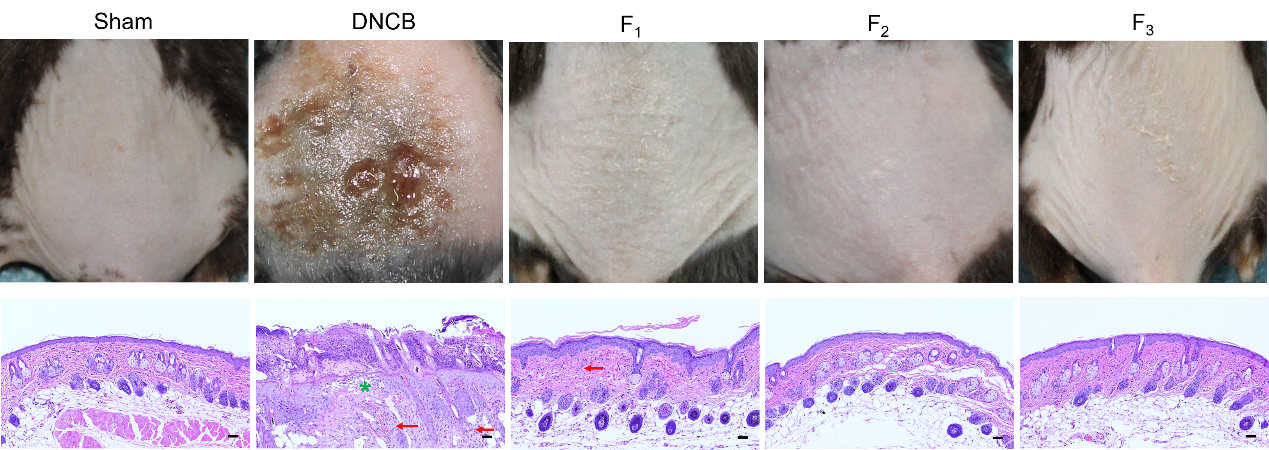


**Fig. S17.** Evaluation of skin irritation treatment with various formula hydrogels (therapeutic dosage: 0.5mL/daily, duration: six days). Photograph and histopathological images of mice back skin in skin irritation study (the green asterisk: confluent nests of apoptotic keratinocytes, the red arrows: perivascular infiltrate of lymphocytes). Scale bars:50 μm, sham: control group, DNCB: 1% 2,4-dinitrochlorobenzene (DNCB) induced contact dermatitis groups, F_1_: Car@MTX hydrogel, F_2_: Car@ZA hydrogel, F_3_: Car@MTX-ZA hydrogel.


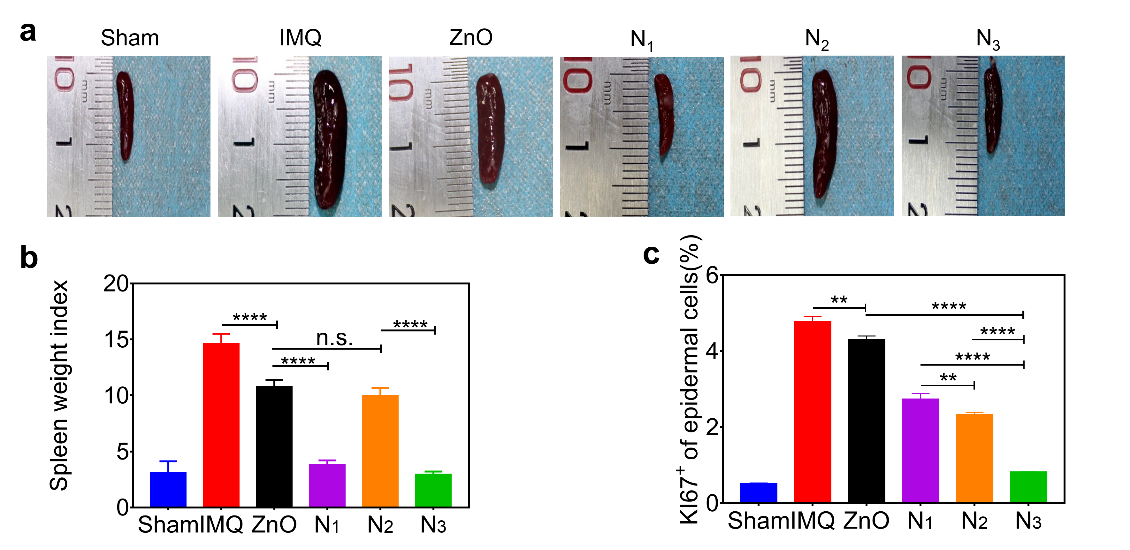


**Fig. S18.** Evaluation of systemic immune responses after various hydrogels treatment (therapeutic dosage: 0.5mL/daily, duration: six days). Representative system inflammatory response was evaluated by spleen length (a) and spleen weight index (b) in IMQ-induced psoriatic mice with or without different formula hydrogels transdermal therapy. (c) Quantitative immunohistochemistry analysis of KI67^+^ in the dorsal skin sections from mice. (n =4, mean ± SEM), ^*^*p*<0.05, ^***^*p*<0.001, ^****^*p*<0.0001 vs. IMQ group,sham: control group, IMQ: imiquimod group, N_1_: Car@NMs@MTX hydrogel, N_2_: Car@NMs@ZA hydrogel, N_3_: Car@NMs@MTX-ZA hydrogel.


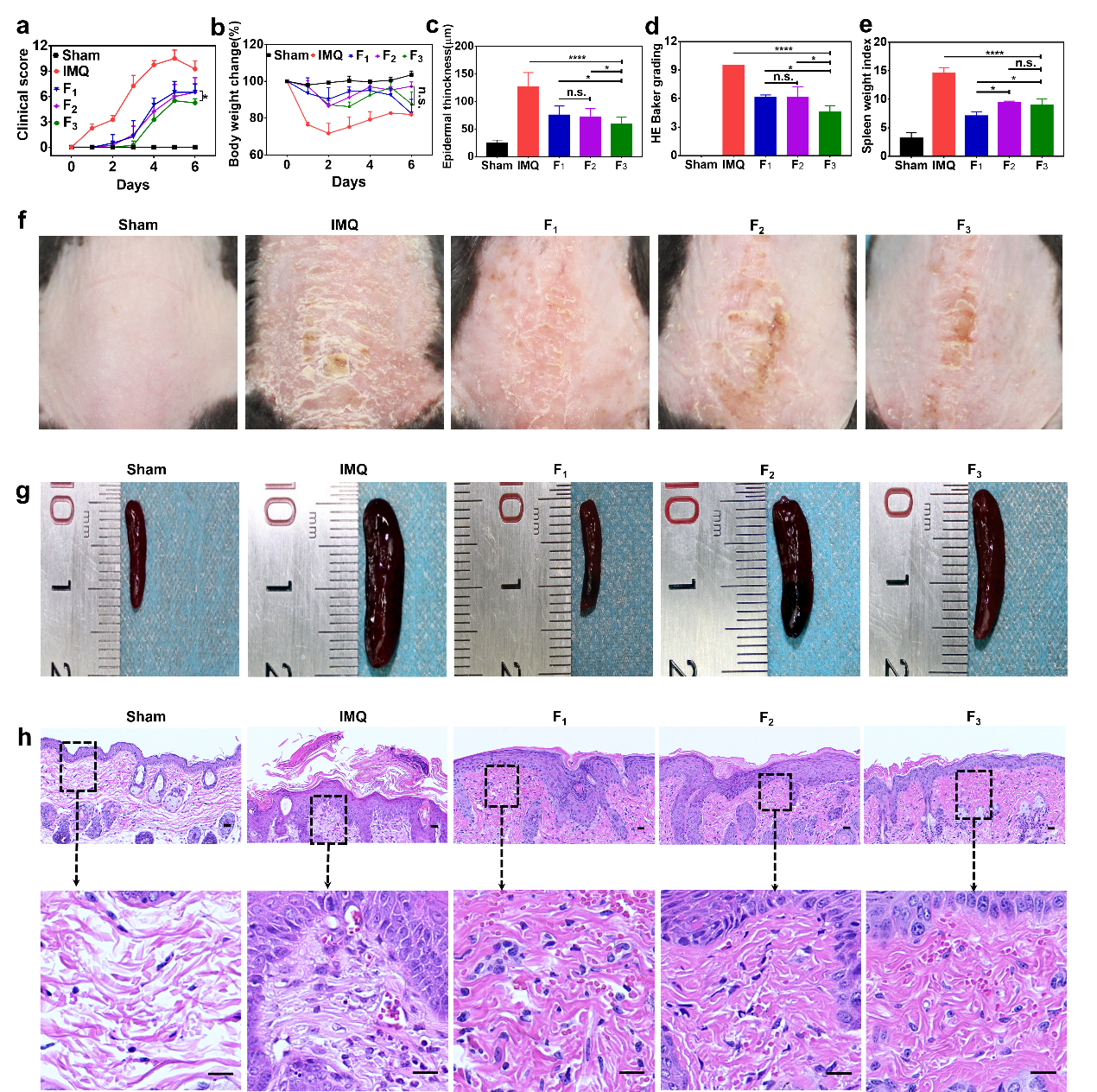


**Fig. S19.** Efficacy of different formula hydrogels on IMQ-induced psoriasiform skin inflammation (therapeutic dosage: 0.5mL/daily, duration: six days). (a) PASI scores of skin lesions were monitored. (b) Percent change of body weight. (c) Quantification of the epidermal thickness, scale bars:50 μm. (d) Histopathological score of the dorsal skin sections from mice stained with H&E, scale bars:50 μm. (e) Evaluation of spleen weight index in IMQ-induced psoriatic mice with or without different formula hydrogels treatments. (f) Representative images of the dorsal skin from mice. (g) Representative system inflammatory response was evaluated by spleen length. (h) Representative H&E images of the dorsal skin sections from mice. n =4, mean ± SEM, scale bars:20 μm, n.s. (not significant), ^*^*p*<0.05, ^***^*p*<0.001, ^****^*p*<0.0001 vs. IMQ group, sham: control group, IMQ: imiquimod group, F_1_: Car@MTX hydrogel, F_2_: Car@ZA hydrogel, F_3_: Car@MTX-ZA hydrogel.


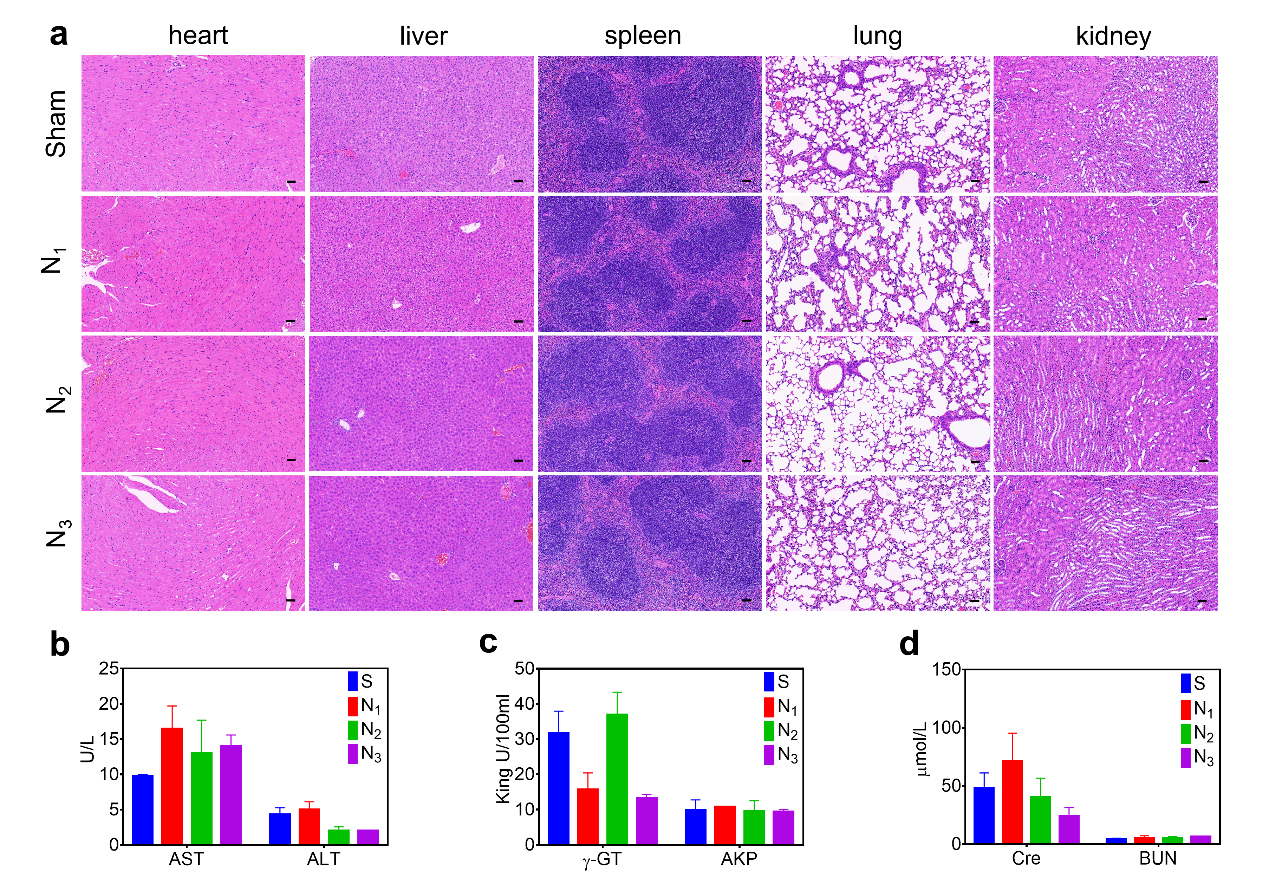


**Fig. S20.** Toxicological studies of different formula hydrogels transdermal therapy on healthy mice (therapeutic dosage: 0.5mL/daily, duration: six days). (a) Representative H&E-stained sections of the main organs of each group. scale bar: 50 μm. (b-d) Evaluation of hepatotoxicity and nephrotoxicity of each group by measuring serum biochemical indicators. S: sham group, N_1_: Car@NMs@MTX hydrogel, N_2_: Car@NMs@ZA hydrogel, N_3_: Car@NMs@MTX-ZA hydrogel.


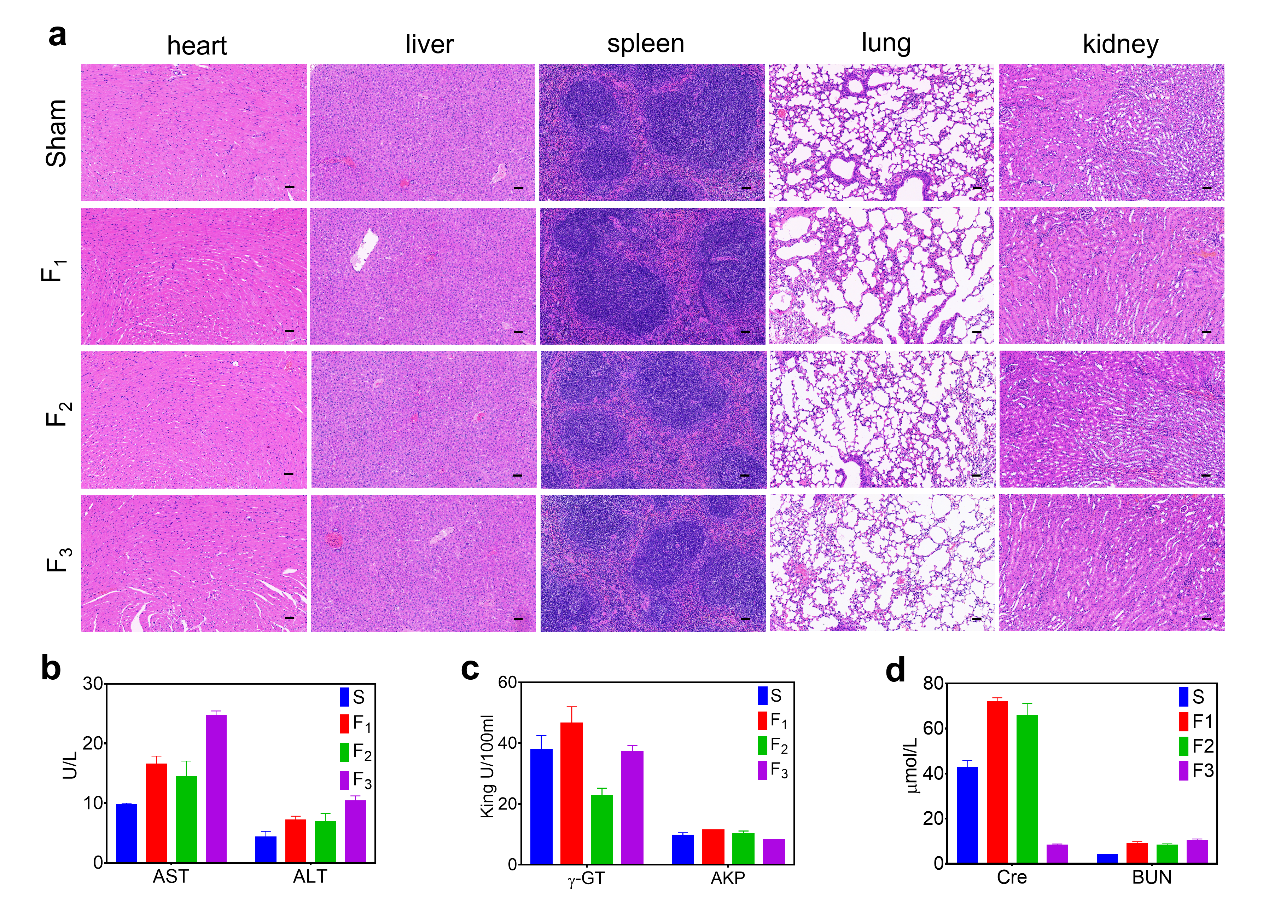


**Fig. S21.** Toxicological studies of different formula hydrogels on healthy mice (therapeutic dosage: 0.5mL/daily, duration: six days). (a) Representative H&E-stained sections of the main organs of each group. scale bar: 50 μm. (b) Evaluation of hepatotoxicity and nephrotoxicity of each group by measuring serum biochemical indicators. S: sham group, F_1_: Car@MTX hydrogel, F_2_: Car@ZA hydrogel, F_3_: Car@MTX-ZA hydrogel.


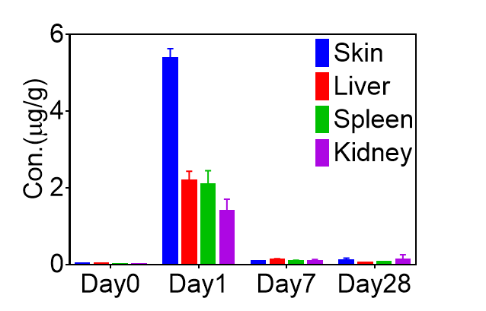


**Fig. S22.** Distribution of Ag^+^ in main internal organs and skin of each group after Car@NMs@MTX-ZA hydrogel treatment at different timepoints.

**Table S1.** Relative amount of Ag ions from three types of ZnO/Ag.

| Materials | Ag in materials  (μg mg^-1^) |
| --- | --- |
| 0.01 wt% ZnO/Ag | 0.05 |
| 0.1 wt% ZnO/Ag | 0.34 |
| 1% wt ZnO/Ag | 5.20 |

**Table S2.** Primers used in qRT-PCR

| Primer name | Forward primer  (5'→3') | Reverse primer  (5'→3') |
| --- | --- | --- |
| β-actin | CCCTGGAGAAGAGCTACGAG | GGAAGGAAGGCTGGAAGAGT |
| TNF-α | CGAGTCTGGGCAGGTCTA | GAAGTGGTGGTCTTGTTGC |
| IL-1β | ATGGCTTATTACAGTGGCA | TAGTGGTGGTCGGAGATT |
| IL-23 | TGAACAACTGAGGGAACCAA | AGCAGCAACAGCAGCATTAC |
| IL-6 | GGGCTCTTCGGCAAATGTAG | TGCCCAGTGGACAGGTTTCT |
| CXCL1 | TCTGGCTTAGAACAAAGGGG | TCCAGTAAAGGTAGCCCTTG |
| S100A7 | CACTCATCCTTCTACTCGTGA | GTTGGGGAAGTTCTCCTTCA |
| DEFB4A | CAGCCATGAGGGTCTTGTAT | AGCATTTTGTTCCAGGGAGA |
| IL-36R | CTTTCTCCTTTCCCGCTG | GCTGGCTTGCTGAAAGTATC |
| IL-17A | GTCCATCTCATAGCAGCAC | TTGTGATTCCTCCTTCACT |
| K17 | CCCTCGGGGGTAGCAG | CATTGAGGTTCTGCATGGTG |

**Table S3.** Histologic Baker grading system for psoriasis

| Microscopic criteria | | Score |
| --- | --- | --- |
| Regular elongation of the rete ridge | 0.5 |  |
|  | 1 |  |
|  | 1.5 |  |
| The infiltration of mononuclear or multinucleate cells in dermis | 0.5 |  |
|  | 1 |  |
|  | 1.5 |  |
| Munro abscesses in corneum | 2 |  |
| [hyperkeratosis](javascript:;) | 0.5 |  |
| parakeratosis | 1 |  |
| Thinning or disappearance of granular layers in the epidermis | 1 |  |
| Acanthosis | 1 |  |
| Elongation of the dermal papillae | 0.5 |  |
| angiotelectasis | 0.5 |  |

**Table S4.** The detailed compositions of various formula hydrogels and corresponding indicators for the evaluation of transdermal therapy efficacy on IMQ-induced psoriasiform skin inflammation (n = 3, mean ± SEM).

| Type | Description | Compositions | MTX  (μg/mL) | Clinical Score | HE baker grading | Epidermal thickness  (μm) | Spleen weight index |
| --- | --- | --- | --- | --- | --- | --- | --- |
| N1 | Car@NMs@MTX hydrogel | PCL-PEG-PCL+MTX | 249.46 | 2.25±0.62 | 5.13±0.52 | 58.83±3.63 | 3.85±0.18 |
| N2 | Car@NMs@ZA hydrogel | PCL-PEG-PCL+ZA |  | 2.46±0.52 | 5.13±0.62 | 55.39±2.65 | 9.99±0.35 |
| N3 | Car@NMs@MTX-ZA hydrogel | PCL-PEG-PCL+ZA | 248.21 | 0.53±0.23 | 3.25±0.15 | 38.95±1.13 | 2.95±0.13 |
| F1 | Car@MTX hydrogel | MTX | 250 | 2.82±1.15 | 6.12±0.13 | 75.49±3.29 | 7.05±0.38 |
| F2 | Car@ZA hydrogel | ZA |  | 2.64±1.08 | 6.13±0.55 | 72.47±2.93 | 9.38±0.15 |
| F3 | Car@MTX-ZA hydrogel | MTX+ZA | 250 | 2.03±0.96 | 4.63±0.31 | 59.30±2.48 | 8.97±0.53 |
| ZnO | ZnO | PCL-PEG-PCL+ZnO |  | 5.10±1.19 | 7.5±0.35 | 111.75±2.99 | 10.80±0.28 |
